# TP53 missense mutation reveals gain-of-function properties in small-sized KRAS transformed pancreatic ductal adenocarcinoma

**DOI:** 10.1186/s12967-023-04742-y

**Published:** 2023-12-01

**Authors:** Yiran Zhou, Jiabin Jin, Yuchen Ji, Jiaqiang Zhang, Ningzhen Fu, Mengmin Chen, Jun Wang, Kai Qin, Yu Jiang, Dongfeng Cheng, Xiaxing Deng, Baiyong Shen

**Affiliations:** 1 Department of General Surgery, Pancreatic Disease Center, Ruijin Hospital, Shanghai Jiao Tong University School of Medicine, 197 Ruijin Er Road, Shanghai, 200025 China; 2 Research Institute of Pancreatic Diseases, Shanghai Jiao Tong University School of Medicine, Shanghai, China; 3 State Key Laboratory of Oncogenes and Related Genes, Shanghai, China; 4 Institute of Translational Medicine, Shanghai Jiao Tong University, Shanghai, China

**Keywords:** Pancreatic ductal adenocarcinoma (PDAC), TP53 mutation subtype, Gain-of-function properties, Survival outcomes

## Abstract

**Background:**

Although the molecular features of pancreatic ductal adenocarcinoma (PDAC) have been well described, the impact of detailed gene mutation subtypes on disease progression remained unclear. This study aimed to evaluate the impact of different TP53 mutation subtypes on clinical characteristics and outcomes of patients with PDAC.

**Methods:**

We included 639 patients treated with PDAC in Ruijin Hospital affiliated to Shanghai Jiaotong University School of Medicine between Jan 2019 and Jun 2021. The genomic alterations of PDAC were analyzed, and the association of TP53 mutation subtypes and other core gene pathway alterations with patients’ clinical characteristics were evaluated by Chi-squared test, Kaplan-Meier method and Cox regression model.

**Results:**

TP53 missense mutation was significantly associated with poor differentiation in KRAS^mut^ PDAC (50.7% vs. 36.1%, P = 0.001). In small-sized (≤ 2 cm) KRAS^mut^ tumors, significantly higher LNs involvement (54.8% vs. 23.5%, P = 0.010) and distal metastic rate (20.5% vs. 2.9%, P = 0.030) were observed in those with TP53 missense mutation instead of truncating mutation. Compared with TP53 truncating mutation, missense mutation was significantly associated with reduced DFS (6.6 [5.6–7.6] vs. 9.2 [5.2–13.3] months, HR 0.368 [0.200–0.677], P = 0.005) and OS (9.6 [8.0-11.1] vs. 18.3 [6.7–30.0] months, HR 0.457 [0.248–0.842], P = 0.012) in patients who failed to receive chemotherapy, while higher OS (24.2 [20.8–27.7] vs. 23.8 [19.0–28.5] months, HR 1.461 [1.005–2.124], P = 0.047) was observed in TP53^missense^ cases after chemotherapy.

**Conclusions:**

TP53 missense mutation was associated with poor tumor differentiation, and revealed gain-of-function properties in small-sized KRAS transformed PDAC. Nonetheless, it was not associated with insensitivity to chemotherapy, highlighting the neoadjuvant therapy before surgery as the potential optimized strategy for the treatment of a subset of patients.

**Supplementary Information:**

The online version contains supplementary material available at 10.1186/s12967-023-04742-y.

## Introduction

Pancreatic ductal adenocarcinoma (PDAC), the classical and the most common subtype of pancreatic cancer, represents increased incidence and mortality rates worldwide [1]. Surgery is the only potential curative option for a relative small proportion of patients detected early before local progression and distal metastasis. However, most patients will experience a recurrence and die within 5 years after surgery [2].

Among the four most common driver genes of PDAC (KRAS, CDKN2A, TP53 and SMAD4), KRAS mutations are the most common presented in up to 90% of PDAC. Currently, the most widely recognized model of PDAC carcinogenesis is the stepwise model [3], in which firstly, KRAS mutations lead to low-grade dysplastic pancreatic intraepithelial neoplasias (PanINs). Secondly, high-grade PanINs and invasive adenocarcinomas are mostly driven by TP53 and/or CDKN2A and/or SMAD4 mutations. Other low-frequency genetic alterations include the alterations of the COMPASS and SWI/SNF family of Trithorax genes which may also interlink with TP53 and cell cycle pathways, and the alterations of other genes related to DNA damage repair pathway, mismatch repair pathway, RNA processing pathway, PI3K-Akt pathway, WNT pathway, NOTCH pathway, Hedgehog pathway and DNA modification pathway etc., [4] as listed in Additional file 4: Table S1_The Detailed Pathways and Genes Related to PDAC Carcinogenesis and Included in the Genetic Analyses Panel.

TP53 mutations occur in 60–80% of PDAC [5]. The majority of TP53 mutations are missense mutations clustered in the regions encoding its central DNA-binding domain, while a smaller but considerable subset result in truncated TP53 proteins expression [4, 6]. It was noted early on that KRAS^LSL.G12D/+^, Trp53^R172H/+^, PdxCre mice (KPC mice) commonly showed an accelerated progression of PDAC with metastases observed in around 80% of the animals, while reduced invasiveness and decreased metastasis were observed in mice bearing a heterozygous Trp53 conditional knockout allele [7–9]. This has been explained by the potential gain-of-function (GOF) properties of TP53 missense mutations compared with other TP53 loss-of-function (LOF) mutations [10]. Indeed, recent studies also reinforced that TP53 missense mutations heightened the transformed KRAS function and they cooperated to drive PDAC invasiveness and metastasis [11, 12].

However, the GOF hypothesis also remains controversial now and then in the context of the large diversity of TP53 mutations [13]. On one hand, there is still a lack of clinical evidences concerning the differences between TP53 GOF and LOF mutations. On the other, it remains to be studied to which extent the GOF properties of TP53 missense mutations extend, because not all the TP53 missense mutations are the same. A recent study compared certain well-referenced TP53 GOF mutations (R175H, R248W, R248Q, R249S, R273H, R273L and R282W) with the rest in advanced and non-resectable PDAC, and found that they were associated with worse survival [14]. However, these subtypes of TP53 mutations only account for about 20% of patients with TP53 mutations, and about 15% of all the patients, while TP53 missense mutations account for 40–45% of all the PDAC patients. So we found it also interesting to study the GOF properties of TP53 missense mutations, even though not all the TP53 missense mutations are GOF mutations. In this study, we aimed to use a clinical database to evaluate the underlying GOF properties of TP53 missense mutations in the Chinese Asian population with PDAC.

## Methods

### Patient cohort

A total of 639 patients diagnosed with classical PDAC and undergoing genetic analysis in the pancreatic center of Shanghai Ruijin Hospital were enrolled in this study. All of them are Chinese Asians. There were 513 patients undergoing curative surgery, and the biopsy of the primary tumor or the metastatic site was performed in the rest 126 patients between Jan 1st, 2019 and Jun 30th, 2021. The rate of patients undergoing curative surgery was high because the genetic analysis was not done in all the patients with locally advanced or metastatic PDAC, and those late-staged patients without genetic analysis were not included. Patients diagnosed pathologically with other subtypes of pancreatic cancer (intraductal papillary mucinous neoplasm or mucinous cystic neoplasm associated with invasive carcinoma, colloid carcinoma, signet ring cell carcinoma, adenosquamous carcinoma, adenocarcinoma with squamous differentiation, squamous carcinoma, undifferentiated carcinoma, acinar cell carcinoma, ampulla carcinoma etc.) were excluded.

The postoperative treatment mainly referred to adjuvant chemotherapy, the schemes of which included FOLFIRINOX (leucovorin/FOL, fluorouracil/F, irinotecan/IRIN, oxaliplatin/OX) and AG (nab-paclitaxel/A, gemcitabine/G) consistent with the schemes of neoadjuvant therapy in PDAC, and included other derived schemes such as FOLFIRINOX plus PARP inhibitor, monotherapy by gemcitabine etc. [15, 16]. Patients undergoing neoadjuvant therapy before surgery were excluded because firstly, the genetic analysis in our center was mostly done after the neoadjuvant therapy and the surgery, instead of before the neoadjuvant therapy. Secondly, the schemes of neoadjuvant therapy included platinum-based FOLFIRINOX which might induce DNA damage and somatic mutations, and we found it hard to distinguish whether these mutations were carcinogenesis-associated or due to neoadjuvant therapy. And thirdly, the analysis in this study was already complex, so we didn’t include patients after neoadjuvant therapy in this study.

The clinical data were collected before and after surgery or biopsy. The tumor size was determined by CT and pathology in resectable cases, and by CT in metastatic cases. The follow-up data of patients undergoing curative surgery were used for survival analysis, and the last follow-up time was April 30th, 2023. The study was approved by the ethics committee of Shanghai Ruijin Hospital and all patients signed informed consent. The flowchart of the study was shown in Additional file 5: Fig. S1.

### Genetic analysis

The genetic analyses were all performed in the clinical laboratory of Shanghai Ruijin Hospital using next-generation sequencing (NGS) as previously described [17]. Briefly, genomic DNA of the pancreatic tumor was extracted from the curative resection specimen or the biopsy specimen. Genomic DNA of peripheral blood lymphocyte was extracted from blood for somatic mutation calling and for germline variant calling if the consent of the latter was obtained.

As shown in Additional file 4: Table S1_The Detailed Pathways and Genes Related to PDAC Carcinogenesis and Included in the Genetic Analyses Panel, the panel included KRAS and other genes related to the RAS-MAPK pathway (ALK, AXL, DDR2, ERBB1-4, FGFR1-4, KDR, KIT, MET, NTRK1-3, PDGFRA and PDGFRB, RET, SYK, CIC, HRAS, NRAS, NF1, ARAF, BRAF, RAF1, MAP2K1-2, MAP3K1, JAK1-3), TP53, genes related to the cell cycle pathway (CDKN2A, CDKN2B, CDKN1B, RB1), the TGFβ pathway (SMAD4, SMAD2, SMAD3, TGFβR1, TGFβR2), the COMPASS family of the Trithorax genes (KDM6A, KMT2A, KMT2C, KMT2D), the SWI/SNF family of the Trithorax genes (ARID1A, ARID1B, ARID2, CEBPA, PBRM1, SMARCA4, SMARCB1), the homologous recombination pathway (ARID1A which also belongs to the SWI/SNF family, ATM, BAP1, BARD1, BLM, BRCA1, BRCA2, BRIP1, CHEK2, FANCA, FANCC, NBN, PALB2, RAD50, RAD51C, RAD51D), the mismatch repair pathway (MLH1, MSH2, MSH6, PMS2), the RNA processing pathway (RBM10, SF3B1, U2AF1), the PI3K-Akt pathway (PTEN, TSC1, TSC2), the WNT pathway (APC, AXIN1, CTNNB1, FAT1, SOX9, RNF43), the NOTCH pathway (NOTCH1, NOTCH2, NOTCH3, NOTCH4), the hedgehog pathway (PTCH1, SUFU), and the DNA modification pathway (MUTYH, DNMT3A, DNMT3B, TET2).

The missense mutation was determined to be deleterious or benign by Polymorphism Phenotyping v2 (PolyPhen-2) system [18]. Frameshift insertion or deletion, nonsense mutation, splice-site mutation and initiation codon mutation were considered to be truncating mutation. Other types of mutation included in-frame insertion or deletion, deletion-insertion mutation, fusion, silent mutation and readthrough mutation, and were considered to be deleterious in this study. The annotations of mutations were listed in Additional file 1: Raw Data 1_Annotations of mutations, and the clinical and genetic information of all the patients were recorded in Additional file 2: Raw Data 2_Information of patients.

### Definition of mutational subtypes of PDAC

Among the 96 single-base substitution (SBS) COSMIC mutational signatures (Version 3.3) based on the prevalence of the six possible base substitutions, three types of signatures predominate in PDAC which are those related to age (SBS 1/clock-like) in the most of cases, to homologous recombination deficiency (HRD) in 10% (SBS 3) and to mismatch repair deficiency (MMRD) in 1–2% (SBS 6, 15, 21, 26 and 44) [3, 19, 20]. In this study, patients were classified as the MMRD subtype if deleterious mutations of the four MMRD genes existed, and as the HRD subtype if one was not the MMRD subtype and that deleterious mutations of the HRD genes were found. The rest of patients were considered as the age-related subtype.

### Statistical analysis

SPSS version 22.0 (IBM) was used for statistical analysis and GraphPad Prism 5 was used for plotting survival curves. Differences between groups were evaluated by the Chi-squared test or Fisher’s exact test according to standard protocols. Briefly, the chi-squared test is considered if the sample size is large when expected counts all exceed 5. The Fisher’s Exact test is used for small samples when the use of chi-squared test is not appropriate, and where cells in the table have expected counts that are less than 5, and/or cell counts are smaller than 20, and/or the column or row marginal values are extremely uneven. The Kaplan–Meier plots and the univariate log-rank test were used for visually demonstrating disease-free survival (DFS) and overall survival (OS). Because the univariate log-rank test was not reliable enough to evaluate the survival differences, we used multivariate Cox regression model instead to compare the survival outcomes between different groups of the Kaplan–Meier plots, after adjusting for comutations and clinical covariates. The results of the multivariate Cox analysis were presented by hazard ratio (HR). A two-sided P value < 0.05 was considered statistically significant.

### Cell culture, transfection and quantitative real-time PCR (qRT-PCR)

Human pancreatic cancer cell lines PANC-1 and CFPAC-1 were obtained from the Cell Bank of the Chinese Academy of Sciences. PANC-1 cells were cultured in DMEM and CFPAC-1 cells were cultured in IMDM supplemented with 10% fetal bovine serum (FBS).

For in vitro experiments, TP53 siRNA (si-TP53) and NC siRNA (si-NC) were synthesized by Bioegene (Shanghai, China). The transfections were performed using Hilymax (Dojindo). Cells were collected 48 h post-transfection. RNA was extracted using TRIzol reagent (Invitrogen, USA). The HiScript III RT SuperMix (TOYOBO, Japan) was used for reverse transcription, and the AceQ Universal SYBR qPCR Master Mix (AG, China) was used to detect the RNA expression levels, which were normalized to β-actin. The divergent and convergent primers of different types of mutant TP53 were used for RT-PCR. The siRNA sequences and the primer sequences are listed in Additional file 4: Table S2_The siRNA Sequences and the Primer Sequences Used in the Study.

### Cell migration assays

The Transwell migration assays were performed as previously described [21]. Briefly, transfected pancreatic cancer cells (5 × 10^4^ cells/100 µL) were suspended in serum-free medium and plated in the top chambers. The lower chambers were filled with 700 µL of DMEM or IMDM supplemented with 10% FBS. After 24 h, the pancreatic cancer cells that migrated from the upper chamber were fixed with 1% crystal violet stain solution for 20 min at room temperature and migrated cells were counted manually, and the relative cell migration percentage was calculated as the ratio of the number of migrated cells to that of the negative control.

For the wound healing assays, wound areas were made using 200 µL pipette tips after 48 h of transfection, and this time point was defined as 0 h. Afterwards, the cells were cultured for 36 h in serum-free medium after plating, and the wound areas were observed and photographed.

## Results

### Mutational landscape of the patient cohort

A total of 639 patients were included in this study and KRAS mutation was present in 595 (93.1%) patients, as shown in Additional file 4: Table S3_Patient Cohort and KRAS Status. 473 KRAS^mut^ patients and 40 KRAS^WT^ patients underwent curative surgery, whose overall clinical information were shown in Additional file 4: Table S4_Clinical Information of the Patient Cohort.

As shown in Additional file 4: Table S5_Core Gene Pathway Alterations in KRAS^mut^ and KRAS^WT^ PDAC, the 595 KRAS^mut^ PDAC included 491 (82.5%) age-related subtype, 92 (15.5%) HRD subtype and 12 (2.0%) MMRD subtype, while the 44 KRAS^WT^ PDAC included 26 (59.1%) age-related subtype, 17 (38.6%) HRD subtype and 1 (2.3%) MMRD subtype. Compared with KRAS^mut^ PDAC, KRAS^WT^ PDAC were more likely to be the HRD subtype (38.6% vs. 15.5%, P < 0.001), showed lower alteration frequencies in the three traditional pathways (TP53, cell cycle and TGFβ), comparable frequencies in Trithorax genes, and higher frequencies in NOTCH pathway, Hedgehog pathway and DNA modification pathway.

### Core gene pathway alterations and association with tumor differentiation in KRAS^mut^ PDAC

The tumor differentiation status of each sample was judged by two independent pathologists from the Department of Pathology of Shanghai Ruijin Hospital with official pathology reports. The differentiation status of PDAC was according to defined WHO criteria, including the presence of tubular structures or solid growth, the presence of mucin, nuclear polymorphism and number of mitoses. Specifically, tumors with more than 30% of areas showing features of poor differentiation were categorized as poor differentiated PDAC. Examples of the histological features of well, moderate and poor differentiated PDAC are shown in Additional file 5: Fig. S2.

As shown in Table 1, among TP53 and other eleven pathways analyzed, TP53 was the only one related to poor tumor differentiation in KRAS^mut^ PDAC. Notably, the rate of TP53 missense mutation in poor differentiated PDAC was 50.7% (vs. 36.1% in moderate and well differentiated PDAC, P = 0.001), while the frequency of TP53 truncating mutation was comparable between the two groups (18.7% vs. 20.4%, P = 0.612). Same results were obtained in patients with age-related PDAC, where the rate of TP53 missense mutation was 50.9% in poor differentiated PDAC (vs. 40.0% in moderate and well differentiated PDAC, P = 0.021), as shown in Additional file 4: Table S6_Core Gene Pathway Alterations and Association with Tumor Differentiation in age-related KRAS^mut^ PDAC.

We also conducted multivariate Logistic regression to validate the influence of core gene pathway alterations on tumor differentiation in KRAS^mut^ PDAC. As shown in Additional file 4: Table S7_Multivariate Logistic Regression of Core Gene Pathway Alterations for Tumor Differentiation in KRAS^mut^ PDAC, TP53 missense mutation was the only alteration related to poor tumor differentiation in multivariate analysis (OR 1.848 [1.290–2.647], P = 0.001).

**Table 1:** Core Gene Pathway Alterations and Association with Tumor Differentiation in KRAS^mut^ PDAC

Mutated pathway in KRAS^mut^ PDAC	Moderate and well differentiated n=260	P	Poor differentiated n=284	P
TP53				
Missense	94 (36.1%)	1	144 (50.7%)	**0.001**^†^
Truncating	53 (20.4%)	1	53 (18.7%)	0.612^†^
Others^*^	2 (0.8%)	1	11 (3.9%)	**0.018**^†^
Overall mutated	149 (57.3%)	1	208 (73.3%)	<0.001†
Cell cycle				
CDKN2A	37 (14.2%)	1	52 (18.3%)	0.199^†^
Overall mutated	42 (16.2%)	1	58 (20.4%)	0.199^†^
TGFb	62 (23.8%)	1	60 (21.1%)	**0.447**^†^
2-3 mutated pathways^**^	63 (24.2%)	1	91 (32.0%)	**0.043**^†^
Trithorax	52 (20.0%)	1	70 (24.6%)	0.194^†^
HRD	39 (15.0%)	1	46 (16.2%)	0.701^†^
MMRD	6 (2.3%)	1	6 (2.1%)	0.877^†^
RNA processing	11 (4.2%)	1	8 (2.8%)	0.370^†^
PI3K-Akt	3 (1.2%)	1	3 (1.1%)	1.000^‡^
WNT	26 (10.0%)	1	22 (7.7%)	0.355^†^
NOTCH	10 (3.8%)	1	10 (3.5%)	0.841^†^
Hedgehog	7 (2.7%)	1	7 (2.5%)	0.867^†^
DNA modification	6 (2.3%)	1	11 (3.9%)	0.295^†^

^*^Other mutation subtypes included mixed cases, in-frame insertion or deletion and silent mutation

^**^Alterations of 2-3 pathways among TP53, cell cycle pathway and TGFb pathway; ^†^Chi-squared test; ^‡^Fisher exact test

### Core gene pathway alterations and association with lymph nodes involvement and distal metastasis according to tumor size in KRAS ^mut^ PDAC

At a median number of 13 lymph nodes examined per patient (range, 5–55), lymph nodes (LNs) involvement in resectable KRAS^mut^ PDAC only negatively correlated with WNT pathway alteration (LNs positive rate: 37.2% in WNT^mut^ cases vs. 54.0% in WNT^WT^ cases, P = 0.036) and Hedgehog pathway alteration (LNs positive rate: 16.7% in Hedgehog^mut^ cases vs. 53.4% in Hedgehog^WT^ cases, P = 0.012), as shown in Table 2 and Additional file 4: Table S8_Other Gene Pathway Alterations and Association with Lymph Nodes Involvement. However, after stratification according to tumor size, we found that in the small-sized group (≤ 2 cm), the LNs involvement rate of patients with TP53 missense mutation remained as high as 54.8% compared with TP53^WT^ cases (vs. 23.5%, P = 0.010). In cases with small-sized tumors, the LNs involvement rate of patients with TP53 truncating mutations (27.8% vs. 23.5% in TP53^WT^ cases, P = 0.736), cell cycle pathway (50.0% vs. 34.7% in cell cycle^WT^ cases, P = 0.310) and TGFβ pathway (41.7% vs. 36.1% in TGFβ^WT^ cases, P = 0.712) alterations was only slightly higher than relevant wild-type cases, without significant differences.

**Table 2:** Core Gene Pathway Alterations and Association with Lymph Nodes Involvement

Mutated pathway	KRAS^mut^		KRAS^mut^		KRAS^mut^		KRAS^mut^		KRAS^WT^	
	Overall, n=473		Tumor size ≤ 2cm, n=84		Tumor size (2, 3cm], n=211		Tumor size > 3cm/T4, n=178		Overall, n=40	
	N1-2	P	N1-2	P	N1-2	P	N1-2	P	N1-2	P
TP53										
WT	90/172 (52.3%)	1	8/34 (23.5%)	1	45/79 (57.0%)	1	37/59 (62.7%)	1	12/30 (25.0%)	1
Missense	106/197 (53.8%)	0.776^†^	17/31 (54.8%)	**0.010**^†^	45/80 (56.3%)	0.928^†^	44/86 (51.2%)	0.169^†^	4/9 (40.4%)	0.812^‡^
Truncating	46/93 (49.5%)	0.656^†^	5/18 (27.8%)	0.736^‡^	23/46 (50.0%)	0.451^†^	18/29 (62.1%)	0.953^†^	1/1	–
Others^*^	6/11 (54.5%)	0.886^†^	1/1	–	4/6	–	1/4	–	None	–
Mutated	158/301 (52.5%)	0.972^†^	23/50 (46.0%)	**0.036**^†^	72/132 (54.5%)	0.732^†^	63/119 (52.9%)	0.216^†^	5/10 (50.0%)	0.580^‡^
Cell cycle										
WT	202/387 (52.2%)	1	25/72 (34.7%)	1	92/170 (54.1%)	1	85/145 (58.6%)	1	15/37 (40.5%)	1
Mutated	46/86 (53.5%)	0.828^†^	6/12 (50.0%)	0.310^‡^	25/41 (61.0%)	0.428^†^	15/33 (45.5%)	0.169^†^	2/3 (66.7%)	0.565^‡^
TGFb										
WT	195/374 (52.1%)	1	26/72 (36.1%)	1	94/170 (55.3%)	1	75/132 (56.8%)	1	15/34 (44.1%)	1
Mutated	53/99 (53.5%)	0.805^†^	5/12 (41.7%)	0.712^‡^	23/41 (56.1%)	0.926^†^	25/46 (54.3%)	0.771^†^	2/6 (33.3%)	1.000^‡^
Mutated pathways										
0-1	180/344 (52.3%)	1	22/68 (32.4%)	1	84/152 (55.3%)	1	74/124 (59.7%)	1	16/35 (45.7%)	1
2-3^**^	68/129 (52.7%)	0.940^†^	9/16 (56.3%)	0.075^†^	33/59 (55.9%)	0.930^†^	26/54 (48.1%)	0.154^†^	1/5 (20.0%)	0.373^‡^
Trithorax										
WT	202/374 (54.0%)	1	27/75 (36.0%)	1	100/166 (60.2%)	1	75/133 (56.4%)	1	10/29 (34.5%)	1
Mutated	46/99 (46.5%)	0.181^†^	4/9 (44.4%)	0.620^‡^	17/45 (37.8%)	**0.007**^†^	25/45 (55.6%)	0.922^†^	7/11 (63.6%)	0.096^‡^
WNT										
WT	232/430 (54.0%)	1	31/78 (39.7%)	1	106/187 (56.7%)	1	95/165 (57.6%)	1	17/38 (44.7%)	1
Mutated	16/43 (37.2%)	**0.036**^†^	0/6	–	11/24 (45.8%)	0.314^†^	5/13 (38.5%)	0.181^†^	0/2	–
Hedgehog										
WT	246/461 (53.4%)	1	31/81 (38.3%)	1	116/204 (56.9%)	1	99/176 (56.3%)	1	16/37 (43.2%)	1
Mutated	2/12 (16.7%)	**0.012**^†^	0/3	–	1/7 (14.3%)	**0.046**^‡^	1/2 (50.0%)	1.000^‡^	1/3 (33.3%)	1.000^‡^

^*^Other mutation subtypes included mixed cases, in-frame insertion or deletion and silent mutation

^**^Alterations of 2-3 pathways among TP53, cell cycle pathway and TGFb pathway; ^†^Chi-squared test; ^‡^Fisher exact test

Interestingly, as shown in Table 2, the LNs involvement rate of large-sized (> 3 cm) resectable tumors with TP53 missense mutation was lower than those without TP53 mutation (LNs positive rate: 51.2% in TP53^missense^ cases vs. 62.7% in TP53^WT^ cases, P = 0.169), though without significant difference. It was also found that in resectable cases, the rate of TP53 missense mutation was higher in large-sized (> 3 cm) tumors (48.3% vs. 36.9% in small-sized tumors, P = 0.083), as shown in Additional file 4: Table S9_Frequency of Core Gene Pathway Alterations according to Tumor Size in KRAS^mut^ PDAC. We assumed that firstly, those with TP53 missense mutation that didn’t metastasize in the early stage were tumors with specifically lower invasiveness, therefore had lower LNs involvement rate. Secondly, if early metastasis hadn’t occurred, KRAS^mut^ PDAC with TP53 missense mutation tended to grow faster. Certainly, these two hypotheses need further validation.

With regard to distal metastasis, the metastatic rate of patients with TP53 mutations (20.4% vs. 17.5%, P = 0.389), cell cycle pathway (20.9% vs. 19.0%, P = 0.642) and Trithorax genes (22.0% vs. 18.6%, P = 0.384) alterations was only slightly higher than relevant wild-type cases, without significant differences, as shown in Table 3 and Additional file 4: Table S10_Other Gene Pathway Alterations and Association with Distal Metastasis. TGFβ pathway alteration was the only potential factor significantly associated with distal metastasis (metastatic rate: 26.8% in TGFβ^mut^ cases vs. 17.1% in TGFβ^WT^ cases, P = 0.011). The impact of TGFβ pathway alteration on distal metastasis seemed widely reported although its detailed characteristics remained controversial [22, 23]. After stratification according to tumor size, patients with TP53 missense mutation instead of truncating mutation showed significant higher metastatic rate than those without TP53 mutation (metastatic rate: 20.5% in KRAS^mut^TP53^missense^ PDAC vs. 2.9% in KRAS^mut^TP53^WT^ PDAC, P = 0.030), indicating that TP53 missense mutation probably led to early invasiveness and metastasis in KRAS^mut^ PDAC patients.

**Table 3:** Core Gene Pathway Alterations and Association with Distal Metastasis

Mutated pathway	KRAS^mut^		KRAS^mut^		KRAS^mut^		KRAS^mut^		KRAS^WT^	
	Overall, n=595		Tumor size ≤ 2cm, n=96		Tumor size (2, 3cm], n=261		Tumor size > 3cm/T4, n=238		Overall, n=44	
	Metastasis	P	Metastasis	P	Metastasis	P	Metastasis	P	Metastasis	P
TP53										
WT	37/212 (17.5%)	1	1/35 (2.9%)	1	16/95 (16.8%)	1	20/82 (24.4%)	1	3/33 (9.1%)	1
Missense	57/257 (22.2%)	0.203^†^	8/39 (20.5%)	**0.030**^‡^	23/103 (22.3%)	0.332^†^	25/114 (21.9%)	0.686^†^	0/9	–
Truncating	20/114 (17.5%)	0.984^†^	2/20 (10.0%)	**0.546**^‡^	10/56 (17.9%)	0.873^†^	8/38 (21.1%)	0.688^†^	1/2	–
Others^*^	1/12 (8.3%)	0.413^‡^	1/2	–	1/7 (14.3%)	0.861^‡^	0/4	–	None	–
Mutated	78/383 (20.4%)	0.389^†^	11/61 (18.0%)	^**0.030‡**^	34/166 (20.5%)	0.472^†^	33/156 (21.2%)	0.568^†^	1/11 (9.1%)	1.000^‡^
Cell cycle										
WT	92/485 (19.0%)	1	10/82 (12.2%)	1	36/206 (17.5%)	1	46/197 (23.4%)	1	4/41 (9.8%)	1
Mutated	23/110 (20.9%)	0.642^†^	2/14 (14.3%)	0.222^‡^	14/55 (25.5%)	0.182^†^	7/41 (17.1%)	0.379^†^	0/3	–
TGFb										
WT	78/457 (17.1%)	1	8/80 (10.0%)	1	34/204 (16.7%)	1	36/173 (20.8%)	1	4/38 (10.5%)	1
Mutated	37/138 (26.8%)	^**0.011†**^	4/16 (25.0%)	0.098^‡^	16/57 (28.1%)	0.053^†^	17/65 (26.2%)	0.377^†^	0/6	–
Mutated pathways										
0-1	75/424 (17.7%)	1	7/75 (9.3%)	1	30/182 (16.5%)	1	38/167 (22.8%)	1	4/39 (10.3%)	1
2-3^**^	40/171 (23.4%)	0.111^†^	5/21 (23.8%)	0.076^‡^	20/79 (25.3%)	0.096^†^	15/71 (21.1%)	0.782^†^	0/5	–
Trithorax										
WT	86/463 (18.6%)	1	9/84 (10.7%)	1	35/201 (17.4%)	1	42/178 (23.6%)	1	2/31 (6.5%)	1
Mutated	29/132 (22.0%)	0.384^†^	3/12 (25.0%)	0.162^‡^	15/60 (25.0%)	0.190^†^	11/60 (18.3%)	0.397^†^	2/13 (15.4%)	0.570^‡^
HRD										
WT	94/503 (18.7%)	1	8/83 (9.6%)	1	43/219 (19.6%)	1	43/201 (21.4%)	1	1/27 (3.7%)	1
Mutated	21/92 (22.8%)	0.355^†^	4/13 (30.8%)	**0.032**^‡^	7/42 (16.7%)	0.654^†^	10/37 (27.0)	0.449^†^	3/17 (17.6%)	0.282^‡^
MMRD										
WT	111/583 (19.0%)	1	12/96 (12.5%)	1	49/257 (19.1%)	1	50/230 (21.7%)	1	4/43 (9.3%)	1
Mutated	4/12 (33.3%)	0.215^‡^	None	–	1/4 (25.0%)	0.575^‡^	3/8 (37.5%)	0.292^‡^	0/1	–

^*^Other mutation subtypes included mixed cases, in-frame insertion or deletion and silent mutation

^**^Alterations of 2-3 pathways among TP53, cell cycle pathway and TGFb pathway; ^†^Chi-squared test; ^‡^Fisher exact test

Similar results were obtained in age-related subtype of KRAS^mut^ PDAC. TP53 missense mutation in small-sized age-related cases was significantly associated with higher LNs involvement rate (LNs positive rate 53.6% in TP53^missense^ cases vs. 25.8% in TP53^WT^ cases, P = 0.029) and distal metastasis (metastatic rate: 17.6% in TP53^missense^ cases vs. 0% in TP53^WT^ cases, P = 0.025), as shown in Additional file 4: Table S11_Core Gene Pathway Alterations and Association with Lymph Nodes Involvement in Age-related PDAC, Additional file 4: Table S12_Frequency of Core Gene Pathway Alterations according to Tumor Size in Age-related KRAS^mut^ PDAC, and Additional file 4: Table S13_Core Gene Pathway Alterations and Association with Distal Metastasis in Age-related PDAC.

### Core gene pathway alterations and association with patients’ survival in resectable KRAS^mut^ cases

**Fig. 1:**
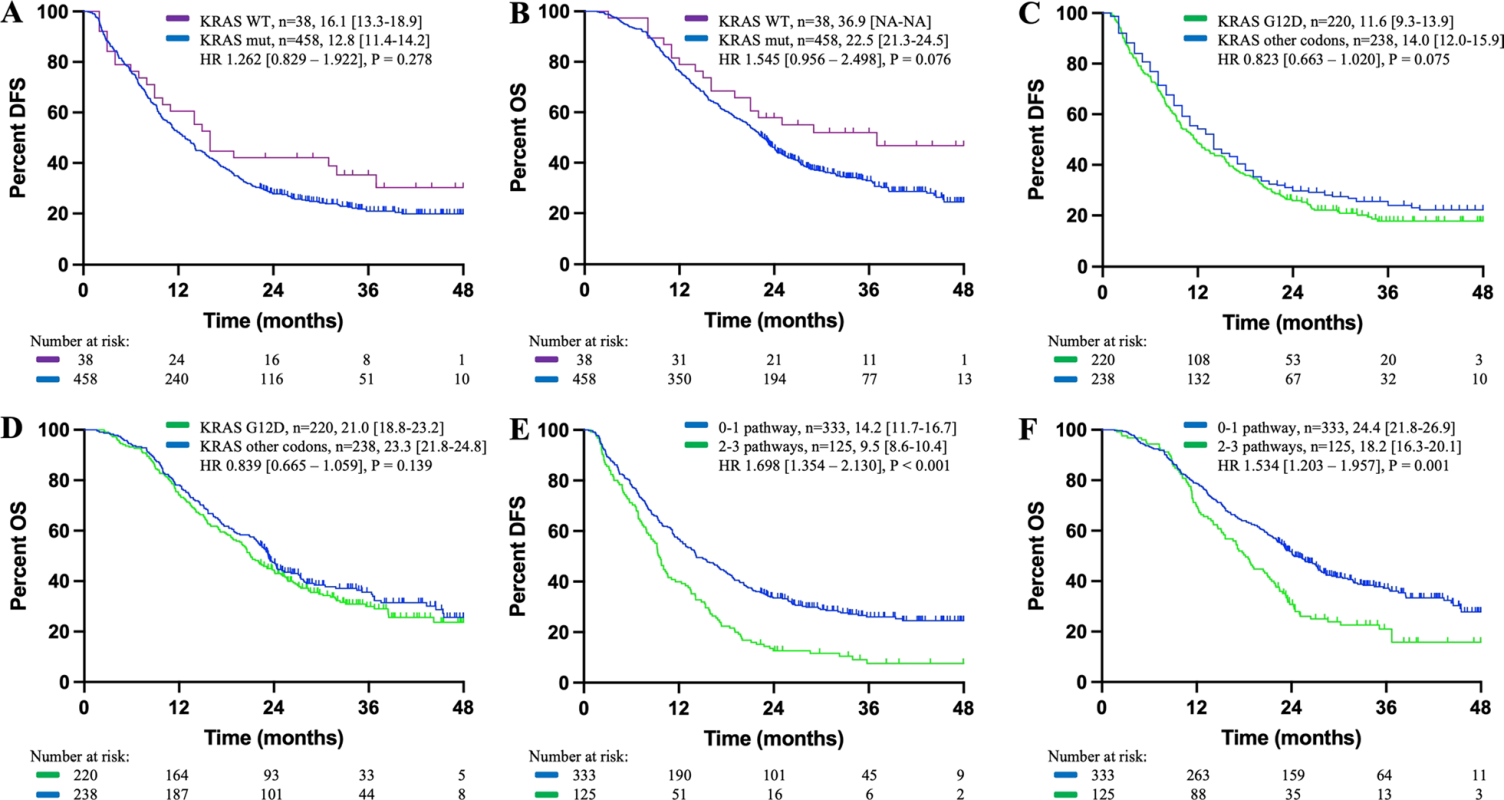
Kaplan-Meier curves for patients (**A**, **B**) with KRAS mutation, (**C**, **D**) with KRAS G12D mutation in KRAS^mut^ cases and (**E**, **F**) with alterations of 2–3 pathways among TP53, cell cycle pathway and TGFβ pathway. DFS and OS were displayed as median [95% CI]. NA indicated not available

The follow-up data of 458 patients with KRAS^mut^ PDAC and 38 patients with KRAS^WT^ PDAC who underwent curative surgery were used for survival analysis. At a median follow-up time of 22.4 months (range, 1.6–52.0 months), reduced DFS (12.8 vs. 16.1 months, HR 1.262 [0.829–1.922], P = 0.278) and OS (22.5 vs. 36.9 months, HR 1.545 [0.956–2.498], P = 0.076) were found in KRAS^mut^ cases compared with KRAS^WT^ cases, as shown in Fig. 1A and B.

As shown in Table 4, the multivariate survival analysis was conducted including 5 mutated pathways, sex, age, vascular invasion, tumor differentiation, tumor size, LNs involvement, resection margin status and adjuvant chemotherapy. It was demonstrated that in patients with KRAS^mut^ PDAC, KRAS^G12D^ mutation was associated with reduced DFS (11.8 vs. 14.0 months, P = 0.075) and OS (21.1 vs. 23.3 months, P = 0.139) compared with mutation of other KRAS codons without significant difference. TP53 mutation was associated with reduced DFS (10.6 months in TP53^mut^ cases vs. 17.3 months in TP53^WT^ cases, P = 0.005) and OS (21.0 months in TP53^mut^ cases vs. 27.0 months in TP53^WT^ cases, P = 0.047), and alterations of cell cycle pathway was also associated with reduced DFS (10.6 months in cell cycle^mut^ cases vs. 13.9 months in cell cycle^WT^ cases, P = 0.017) and OS (19.2 months in cell cycle^mut^ cases vs. 23.3 months in cell cycle^WT^ cases, P = 0.029) with significant difference. Alterations of TGFβ pathway was associated with reduced DFS and OS without significant difference, and that DFS and OS were comparable between patients with and without Trithorax genes alterations. The Kaplan–Meier curves for patients with KRAS^G12D^ mutation and with several pathway mutations were shown in Fig. 1C, D and E F, with HR calculated by Cox regression model after adjusting for comutations and clinical covariates.

**Table 4:** Core Gene Pathway Alterations and Association with Patients’ Survival in Resectable KRAS^mut^ PDAC

Mutated pathwayn and other factors	Number	Disease-free survival in KRAS^mut^ (n=458)			Overall survival in KRAS^mut^ (n=458)		
		Median (mo)	HR (95% CI)	P	Median (mo)	HR [95% CI]	P
KRAS							
G12D	220 (48.0%)	11.83 [9.65–14.01]	1 [Reference]	1	21.07 [18.49–23.65]	1 [Reference]	1
Other codon	238 (52.0%)	13.97 [11.93–16.01]	0.823 [0.663–1.020]	0.075	23.30 [21.79–24.81]	0.839 [0.665–1.059]	0.139
TP53							
WT	164 (35.8%)	17.30 [14.88–19.72]	1 [Reference]	1	26.97 [20.98–32.96]	1 [Reference]	1
Mutated	294 (64.2%)	10.60 [9.14–12.06]	1.390 [1.102–1.752]	**0.005**	21.03 [18.54–23.52]	1.287 [1.003–1.651]	**0.047**
Cell cycle							
WT	376 (82.1%)	13.90 [11.70–16.10]	1 [Reference]	1	23.30 [21.19–25.41]	1 [Reference]	1
Mutated	82 (17.9%)	10.60 [7.97–13.23]	1.388 [1.059–1.818]	**0.017**	19.23 [16.33–22.13]	1.385 [1.034–1.855]	**0.029**
TGFb							
WT	365 (79.7%)	13.63 [11.54 – 15.72]	1 [Reference]	1	23.23 [21.19–25.27]	1 [Reference]	1
Mutated	93 (20.3%)	10.07 [7.01 - 13.13]	1.201 [0.929–1.552]	0.162	18.77 [14.42–23.12]	1.165 [0.883–1.537]	0.280
Trithorax							
WT	362 (79.0%)	12.93 [10.96 - 14.90]	1 [Reference]	1	22.43 [20.54–24.32]	1 [Reference]	1
Mutated	96 (21.0%)	12.83 [9.76 - 15.90]	0.917 [0.705–1.193]	0.519	23.10 [20.18–26.02]	0.903 [0.656–1.243]	0.532
Male	273 (59.6%)		1.198 [0.966–1.485]	0.099		1.153 [0.912–1.458]	0.233
Age > 70y	108 (23.6%)		1.070 [0.814–1.407]	0.627		1.061 [0.785–1.435]	0.699
Vascular invasion	159 (34.7%)		1.209 [0.952–1.537]	0.120		1.243 [0.962–1.604]	0.096
Poor differentiated	228 (49.8%)		1.573 [1.270–1.949]	**<0.001**		1.645 [1.303–2.076]	**<0.001**
Tumor >3cm	170 (37.1%)		1.439 [1.139–1.816]	**0.002**		1.336 [1.046–1.706]	**0.020**
N1-2	240 (52.4%)		1.412 [1.140–1.749]	**0.002**		1.497 [1.189–1.885]	**0.001**
R1 margin status	72 (15.7%)		1.211 [0.902–1.625]	0.203		1.395 [1.024–1.899]	**0.035**
Chemotherapy	329 (71.8%)		0.571 [0.443–0.736]	**<0.001**		0.042 [0.304–0.531]	**<0.001**

### The impact of TP53 missense mutation on patients’ survival

**Fig. 2:**
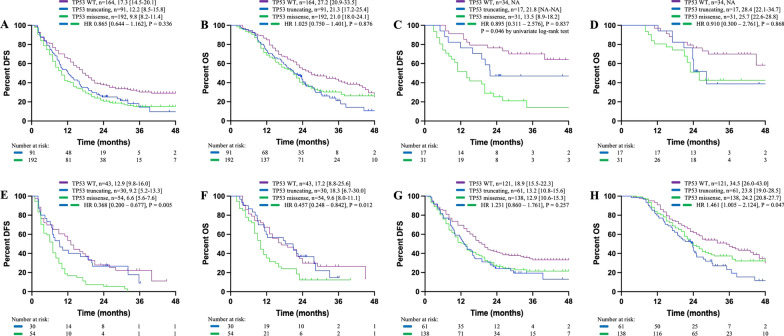
Kaplan–Meier curves for patients with TP53 missense or truncating mutation (**A**, **B**) in resectable cases, (**C**, **D**) in patients with small-sized tumor, (**E**, **F**) in patients who failed to receive chemotherapy and (**G**, **H**) in patients who received chemotherapy. DFS and OS were displayed as median [95% CI]. NA indicated not available

DFS or OS were comparable in patients with TP53 missense and truncating mutation (Fig. 2A and B). In patients with small-sized KRAS^mut^ PDAC, TP53 missense mutation was associated with reduced DFS (13.5 months in TP53^missense^ cases vs. 21.8 months in TP53^truncating^ cases, P = 0.046 by univariate log-rank test, HR 0.895 [0.311–2.576], P = 0.837 by Cox regression analysis) instead of OS (25.7 months in TP53^missense^ cases vs. 28.4 months in TP53^missense^ cases, HR 0.910 [0.300–2.761], P = 0.868), suggesting the potential early micrometastases in these cases (Fig. 2C and D).

However, the impact of TP53 mutation subtypes on patients’ survival remained controversial. Although TP53 missense mutation induced potential early metastases, TP53 truncating mutation was reported to more negatively correlated with patients’ survival than TP53 missense mutation, [24, 25] so we then evaluated whether this was due to tumor’s sensitivity to chemotherapy. Interestingly, TP53 missense mutation was significantly associated with reduced DFS (6.6 months in TP53^missense^ cases vs. 9.2 months in TP53^truncating^ cases, HR 0.368 [0.200–0.677], P = 0.005) and reduced OS (9.6 months in TP53^missense^ cases vs. 18.3 months in TP53^truncating^ cases, HR 0.457 [0.248–0.842], P = 0.012) in patients who failed to receive adjuvant chemotherapy (Fig. 2E, F). In patients who received chemotherapy, while DFS were comparable between the two groups (12.9 months in TP53^missense^ cases vs. 13.2 months in TP53^truncating^ cases, HR 1.231 [0.860–1.761], P = 0.257), TP53 missense mutation was even associated with slightly extended OS compared with tumors with TP53 truncating mutation (24.2 months in TP53^missense^ cases vs. 23.8 months in TP53^truncating^ cases, HR 1.461 [1.005–2.124], P = 0.047), as shown in Fig. 2G, H. The decreased survival differences between the missense and truncating group and the increased differences between the wild-type and truncating group in patients who received adjuvant chemotherapy induced us to hypothesize that tumors with TP53 missense mutation were not associated with insensitivity to chemotherapy, compared with tumors with TP53 truncating mutation.

### The impact of the Trithorax gene alterations on patients’ survival according to TP53 mutation status

The Trithorax gene encodes a large family of proteins which serve as active epigenetic regulators counteracting the repressive gene expression programmes guided by the the Polycomb group of proteins [26]. The transcriptional repression of the locus INK4A/ARF downstream of TP53-Rb signaling axis is a potential target of the Polycomb group of proteins, thus alterations of the Trithorax genes might result in the repression of TP53 function, [27] as illustrated in Additional file 5: Fig. S3.

**Fig. 3:**
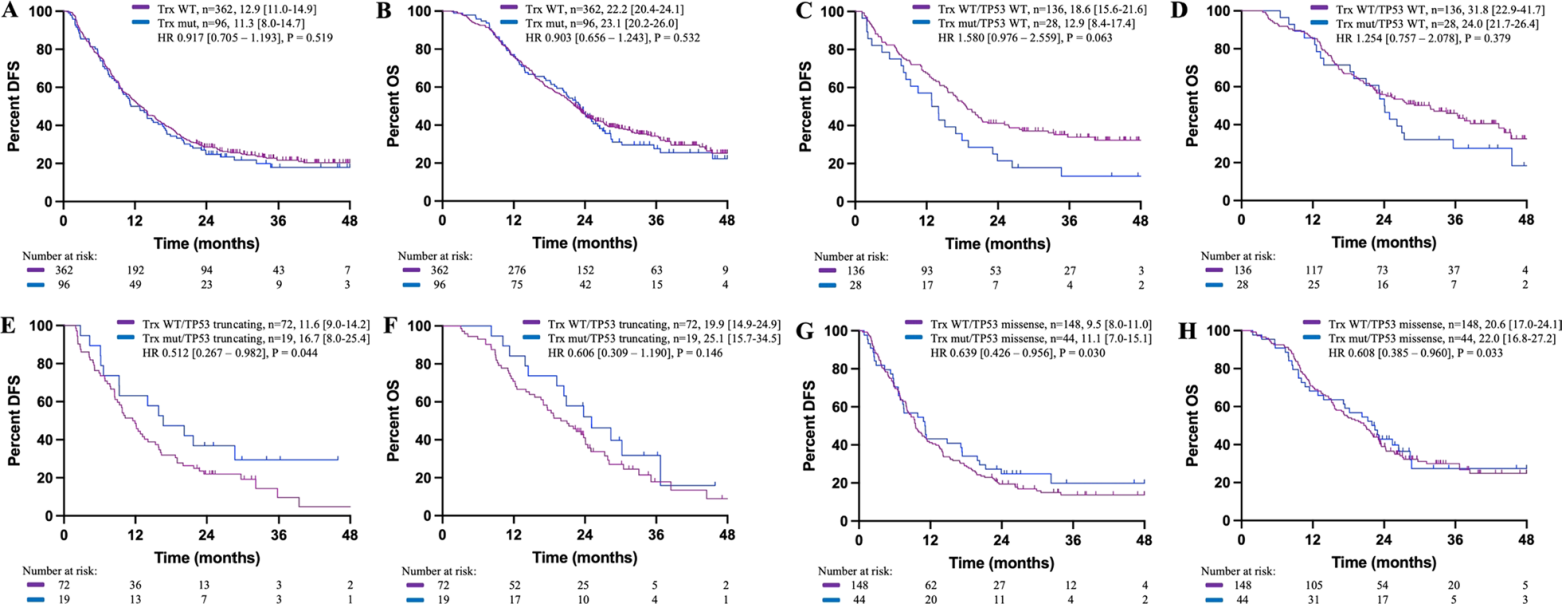
Kaplan–Meier curves for patients with Trithorax genes alterations (**A**, **B**) in resectable cases, (**C**, **D**) in TP53^WT^ cases, (**E**, **F**) in patients with TP53 truncating mutation and (**G**, **H**) in patients with TP53 missense mutation. DFS and OS were displayed as median [95% CI].

As the Trithorax genes were reported to be altered in less frequent but up to 10% of PDAC and that some of its components like KDM6A and ARID1A might influence the development, differentiation and metastasis of PDAC, [4, 28–30] we analyzed the relationship between the Trithorax genes alterations and the survival of patients with different TP53 mutation status. As shown in Fig. 3, although the survival outcomes between patients with and without Trithorax genes alterations were comparable (Fig. 3A and B), Trithorax genes alterations contributed to reduced DFS (12.9 months in Trx^mut^ cases vs. 18.6 months in Trx^WT^ cases, HR 1.580 [0.976–2.559], P = 0.063) and OS (24.0 months in Trx^mut^ cases vs. 31.8 months in Trx^WT^ cases, HR 1.254 [0.757–2.078], P = 0.379) in KRAS^mut^, TP53^WT^ PDAC patients (Fig. 3C and D), higher DFS (16.7 months in Trx^mut^ cases vs. 11.6 months in Trx^WT^ cases, HR 0.512 [0.267–0.982], P = 0.044) and OS (25.1 months in Trx^mut^ cases vs. 19.9 months in Trx^WT^ cases, HR 0.606 [0.309–1.190], P = 0.146) in KRAS^mut^, TP53^truncating^ PDAC patients (Fig. 3E, F), and slightly higher DFS (11.1 months in Trx^mut^ cases vs. 9.5 months in Trx^WT^ cases, HR 0.639 [0.426–0.956], P = 0.030) and OS (22.0 months in Trx^mut^ cases vs. 20.6 months in Trx^WT^ cases, HR 0.608 [0.385–0.960], P = 0.033) in KRAS^mut^, TP53^missense^ PDAC patients (Fig. 3G, H), indicating the different properties between TP53 missense and truncating mutations.

### Validation by Independent TCGA cohort and functional experiments

We managed to validate our conclusions in two ways. Firstly, we used a cohort from the TCGA database including 126 pancreatic cancer patients to validate the association of TP53 mutation status with tumor differentiation. Secondly, we conducted functional experiments in vitro to validate the invasive properties of TP53 missense mutations.

We collected a cohort from the TCGA database with 126 pancreatic cancer patients who had recorded clinical information and SNV analysis, as recorded in Additional file 3: Raw Data 3_Information of the TCGA cohort. There are 93 KRAS^mut^ cases and 33 KRAS^WT^ cases. As shown in Additional file 4: Table S14_Core Gene Alterations and Association with Tumor Differentiation in KRAS^mut^ PDAC in the TCGA cohort, TP53 missense mutation was presented in 0 (0%) well differentiated tumors, while the rate was 54.0% in moderate differentiated tumors (P = 0.001) and 46.7% in poor differentiated tumors (P = 0.007). On the contrary, TP53 truncating mutation was presented in 72.7% well differentiated tumors vs. 20.0% in moderate differentiated tumors (P = 0.001) and 33.3% in poor differentiated tumors (P = 0.036), indicating TP53 missense mutation instead of truncating mutation was associated with poor tumor differentiation.

Notably, tumors with more than 30% of areas showing features of poor differentiation were categorized as poor differentiated PDAC in our study. As shown in Additional file 4: Table S14_Core Gene Alterations and Association with Tumor Differentiation in KRASmut PDAC in the TCGA cohort, there seemed to be more moderate differentiated tumors in the TCGA cohort, probably because that PDAC was defined as moderate differentiated in our hospital only when there was less than 30% of areas showing features of poor differentiation.

**Fig. 4:**
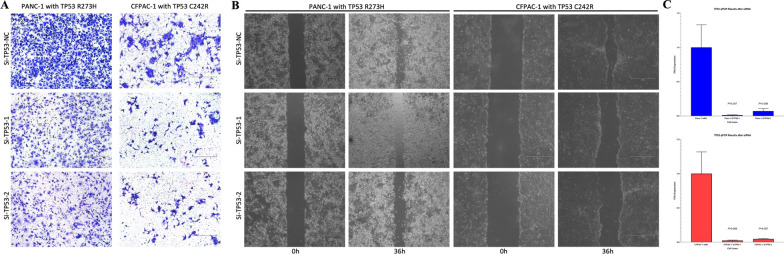
TP53 knock-out was associated with lower invasiveness compared with TP53 missense mutations. **A** Transwell migration assay of PANC-1 and CFPAC-1 cells after knock-out of the TP53 missense mutation. **B** Wound healing assays performed using PANC-1 and CFPAC-1 cells after knock-out of the TP53 missense mutation. **C** Validation of the knock-out of the TP53 missense mutation by qRT-PCR.

Since the volume of the TCGA cohort was not large enough to validate the invasive properties of TP53 missense mutations, we next used two pancreatic cancer cell lines both with KRAS and TP53 missense mutation to perform functional experiments. One cell line was PANC-1 carrying a heterozygous KRAS^G12D^ allele (c.35G > A) and a heterozygous TP53^R273H^ allele (c.818G > A). The other cell line was CFPAC-1 carrying a heterozygous KRAS^G12V^ allele (c.35G > T) and a heterozygous TP53^C242R^ allele (c.724T > C). A non-specific si-TP53 was transfected into the two cell lines to knock out the TP53 missense mutants, mimicking TP53 truncating mutations. As shown in Fig. 4, the Transwell migration assays (Fig. 4A) and the wound healing assays (Fig. 4B) showed that TP53 knock-out was associated with lower invasiveness compared with TP53 missense mutations. Using specific primers, qRT-PCR was performed to validate the knock-out of TP53 missense allele by non-specific si-TP53 (Fig. 4C).

## Discussion

As shown inAdditional file 5: Fig. S4, the genomic alterations of PDAC involve different key pathways, which may also interplay one with another. It remained to be an intriguing question to which extent KRAS^mut^ and KRAS^WT^ PDAC varies one from the other. It was believed that RAS-MAPK pathway activation was still a crucial molecular driver of PDAC on condition that KRAS itself was not mutated [31]. Similarly in our study, 14 out of 44 (31.8%) KRAS ^WT^ PDAC bore a deleterious alteration of RAS-MAKP pathway other than KRAS. Alterations of other pathways also potentially contributed to the development of KRAS^WT^ PDAC, such as HRD pathway, NOTCH pathway, Hedgehog pathway and DNA modification pathway.

As for 595 KRAS^mut^ PDAC, 92 (15.5%) cases were classified as the HRD subtype. Some trivial facts reported by Golan T et al. were also found in our study [32]. Firstly, in KRAS^mut^ cases, TP53 was more likely to be intact in HRD subtype than in age-related subtype (mutation rate 52.2% vs. 66.6%, Additional file 4: Table S2). Secondly, TP53 mutation was more common in BRCA1 compared with BRCA2 inactivated cases (87.5% vs. 60.0%). Unlike the HRD subtype whose classification remained various and controversial, the MMRD subtype involved four genes and accounted for 2.0% of cases in our cohort, which was exactly the ratio reported by other groups [19, 33].

It was well established that PDAC could be classified into classical and basal-like/squamous subtypes according to the tumor’s transcriptome, while the latter tended to be more aggressive with increased metastatic potential [31, 34, 35]. Although not completely exclusive, the broadly defined TP53 mutation was to some extent associated with the basal-like subtype and tumor invasiveness [36, 37]. However, the outcomes of patients were usually more associated with tumors’ clinical and pathologic rather than molecular features [38]. In this study, we found that TP53 missense instead of truncating mutation was associated with poor differentiated PDAC, which was an important clinical prognostic factor. CDKN2A mutations have also been shown to promote undifferentiated PDAC in mouse models [34, 39]. Both in our cohort and in the TCGA cohort, however, we found that the alteration of the cell cycle pathway or CDKN2A itself was a less important factor than TP53 missense mutation to promote poor tumor differentiation, as shown in Table 1, Additional file 4: Table S7_Multivariate Logistic Regression of Core Gene Pathway Alterations for Tumor Differentiation in KRAS^mut^ PDAC, and Additional file 4: Table S14_Core Gene Alterations and Association with Tumor Differentiation in KRAS^mut^ PDAC in the TCGA cohort.

The cross-talk of different core gene pathways and its role in clinical outcomes, such as tumor differentiation, are complex in PDAC. For instance, the cell cycle pathway is also downstream of TP53 and Trithorax genes may interlink with TP53 and cell cycle pathways, as listed in Additional file 4: Table S1_The Detailed Pathways and Genes Related to PDAC Carcinogenesis and Included in the Genetic Analyses Panel. Form both univariate and multivariate analysis in our study, TP53 missense mutation was the only alteration significantly related to poor tumor differentiation. Interestingly, however, the two pathways which may interlink with TP53 were also related to poor tumor differentiation (cell cycle: 20.4% in poor differentiated PDAC vs. 16.2% in others; Trithorax: 24.6% in poor differentiated PDAC vs. 20.0% in others) yet without significant differences, as shown in Table 1 and Additional file 4: Table S7_Multivariate Logistic Regression of Core Gene Pathway Alterations for Tumor Differentiation in KRAS^mut^ PDAC. This might probably be explained by the cross-talk between TP53 and the two pathways. And for sure, the clinical outcomes of PDAC are results of the comprehensive interaction of different pathways. To be noted, potential alterations of GATA6 and MYC were not included in this study, because it is the copy number variation instead of the genetic mutation of these two genes that might influence the PDAC development and progression [31].

The GOF hypothesis involved the interaction between TP53, which mutated in its DNA binding domain, and other transcriptional regulators inducing gene expression modifications [40, 41]. It was recently further confirmed that compared with truncating mutations, TP53 missense mutations presented significantly greater cis-effect with higher TP53 protein expression and TP53-S315 phosphosite expression, while the trans-effects were similar between the two groups [42]. To our knowledge, we firstly described that under curative surgery along with adequate LNs resection and pathological examination, TP53 missense mutation was associated with higher LNs involvement rate in small-sized KRAS transformed PDAC. The distal metastatic rate was also significantly higher in small-sized tumors with TP53 missense mutation, which was in accordance with what was observed in genetically engineered mouse models [7–9]. Although it remains an open question whether and how LN metastasis plays an active role in shaping distant metastasis, [43] both of them indicate tumors’ invasiveness and the GOF properties of TP53 missense mutation. However, it was also true that when the tumor got bigger, the GOF properties of TP53 missense mutation became less obvious.

While the age-related subtype predominates in PDAC, the HRD subtype and the MMRD subtype are, to some extent, special PDAC subtypes [3]. Talia Golan et al. reported that the survival outcomes were comparable between the HRD subtype group and non-HRD group in resectable PDAC. However, the HRD status was predictive of platinum response and superior survival in advanced PDAC [32]. As for the MMRD subtype, Robert C Grant et al. found that MMRD-PDACs presented higher tumour mutational burden, were less likely to have mutations in KRAS and SMAD4, were more likely to have basal-like transcriptional programmes yet had longer OS after surgery [33]. As a result, the clinical manifestations of the MMRD subtype are different from the majority of PDAC, where basal-like tumors tend to be more aggressive with worse survival outcomes, as mentioned early in the discussion. Considering the potential differences among the three mutational subtypes of PDAC, we also investigated in this study whether same results were obtained in age-related PDAC to shed more solid evidence. Indeed, it was found that TP53 missense mutation was associated with poor differentiation in age-related KRAS^mut^ PDAC (Additional file 4: Table S6_Core Gene Pathway Alterations and Association with Tumor Differentiation in age-related KRAS^mut^ PDAC). And in small-sized (≤ 2 cm) age-related KRAS^mut^ tumors, TP53 missense mutation was associated with higher LNs involvement (Additional file 4: Table S11_Core Gene Pathway Alterations and Association with Lymph Nodes Involvement in Age-related PDAC) and distal metastic rate (Additional file 4: Table S13_Core Gene Pathway Alterations and Association with Distal Metastasis in Age-related PDAC).

The impact of the four driver genes’ mutation on patients’ outcomes have been studied since a decade ago [44, 45]. Qian ZR et al. reported the significant association of KRAS, especially KRAS^G12D^ mutation, and CDKN2A loss with DFS and OS, and of TP53 mutation with DFS only in resectable PDAC [24]. In this study, we evaluated the impact of genomic features on patients’ outcomes according to gene pathways, and found that in resectable KRAS^mut^ PDAC cases, KRAS^G12D^ and mutations of TP53, cell cycle and TGFβ pathways were all associated with poor survival, while the impact of TP53 and cell cycle pathway mutations turned out to be statistically significant after the multivariate analysis.

McIntyre CA et al. reported that TP53 truncating mutation predicted worse OS than TP53 missense mutation in resectable cases [25]. And a recent study showed that certain well-referenced TP53 GOF mutations (R175H, R248W, R248Q, R249S, R273H, R273L and R282W) were associated with worse survival in advanced and non-resectable PDAC [14]. In our study, we analyzed the survival difference between patients with missense mutations and truncating mutations. Instead of focusing on well-referenced TP53 GOF mutations, we mainly studied the GOF properties of all the patients with TP53 missense mutations. Firstly, the DFS and OS between the TP53 missense and truncating mutation group were comparable, except that TP53 missense mutation was associated with reduced DFS in small-sized PDAC, implying the potential early micrometastases in these cases. Secondly, TP53 missense mutation was associated with reduced DFS and OS than truncating mutation in patients who failed to receive adjuvant chemotherapy, probably due to its GOF properties. Thirdly, the impact of Trithorax genes alterations on patients’ outcomes varied one from another according to the TP53 mutation status. All these facts served as clinical evidences indicating the different properties between TP53 missense and truncating mutations, which might offer help to the potential targeted treatment against TP53 mutations in the future.

It was widely acknowledged that no matter what the mutation subtype is, TP53 mutations may lead to chemotherapy resistance compared with TP53^WT^ cases, because the chemotherapy seems to be highly dependent on the apoptosis-inducing ability of TP53 [10]. Nonetheless, we still found in this study that the adjuvant chemotherapy extended DFS and OS in TP53^missense^ cases (DFS from 6.6 to 12.9 months, OS from 9.6 to 24.2 months) and in TP53^truncating^ cases (DFS from 9.2 to 13.2 months, OS from 18.3 to 23.8 months), indicating that the adjuvant chemotherapy may provide better survival both in TP53^mut^ and TP53^WT^ cases. Although after multivariate Cox analysis, TP53 missense mutation was an independent factor associated with better OS compared with TP53 truncating mutation in patients receiving adjuvant chemotherapy (24.2 months in TP53^missense^ cases vs. 23.8 months in TP53^truncating^ cases, HR 1.461 [1.005–2.124], P = 0.047), the OS of the TP53 missense group was only 0.4 months more than the truncating group. So we neither concluded that the chemotherapy would give better survival rate scenario in TP53^missense^ group than in TP53^truncating^ group, nor hypothesized that TP53 missense mutation was more sensitive to chemotherapy compared with TP53 truncating mutation. Instead, we found it more rational that TP53 missense mutation was not associated with insensitivity to chemotherapy compared with TP53 truncating mutation.

It was recently reported that the neoadjuvant therapy substantially ameliorated the 5-year OS rate compared to upfront surgery (20.5% vs. 6.5%) [46]. According to this study, PDAC with TP53 missense mutations tend to generate local LNs involvement and distal micrometastases in the early stage and are no less sensitive to chemotherapy. Besides, the majority of TP53 mutations in PDAC are the missense subtype [4, 10]. This might probably explain the significant higher 5-year OS rate in patients receiving neoadjuvant therapy, highlighting the chemotherapy before surgery as the potential optimized strategy for the treatment of a subset of patients with PDAC. In clinical practice, further study can be focused on the necessitation of neoadjuvant therapy in resectable PDAC according to patients’ mutational signatures, such as the different TP53 mutation subtypes. To achieve this, the biopsy of the tumor should be primarily done to perform genetic analysis, and relative randomized controlled trials can be undertaken in different patient subgroups with distinct TP53 mutation subtypes.

Some limitations existed in this study. Firstly, only a relative small subset of patients were operated with small-sized pancreatic tumor. Secondly, patients were only analysed based on the genomic features concerning core gene pathways instead of the multi-omic data, and the tumor heterogeneity was not taken into great consideration. Thirdly, the copy number variations including the loss of heterozygosity status of the TP53 mutations were not evaluated in this study.

## Conclusions

TP53 missense mutation was associated with poor tumor differentiation, and revealed gain-of-function properties as it was predictive of early LNs involvement and distal metastasis. Nonetheless, it was not associated with insensitivity to chemotherapy, highlighting the neoadjuvant therapy before surgery as the potential optimized strategy for the treatment of a subset of patients.

### Supplementary information

**Additional file 1.** Raw Data 1_Annotations of mutations.

**Additional file 2.** Raw Data 2_Information of patients.

**Additional file 3.** Raw Data 3_Information of the TCGA cohort.

**Additional file 4: Table S1–S14.** **Table S1.** The Detailed Pathways and Genes Related to PDAC Carcinogenesis and Included in the Genetic Analyses Panel. **Table S2.** The siRNA Sequences and the Primer Sequences Used in the Study. **Table S3.** Patient Cohort and KRAS Status. **Table S4.** Clinical Information of the Patient Cohort. **Table S5.** Core Gene Pathway Alterations in KRAS^mut^ and KRAS^WT^ PDAC. **Table S6.** Core Gene Pathway Alterations and Association with Tumor Differentiation in age-related KRAS^mut^ PDAC. **Table S7.** Multivariate Logistic Regression of Core Gene Pathway Alterations for Tumor Differentiation in KRAS^mut^ PDAC. **Table S8.** Other Gene Pathway Alterations and Association with Lymph Nodes Involvement. **Table S9.** Frequency of Core Gene Pathway Alterations according to Tumor Size in KRAS^mut^ PDAC. **Table S10.** Other Gene Pathway Alterations and Association with Distal Metastasis. **Table S11.** Core Gene Pathway Alterations and Association with Lymph Nodes Involvement in Age-related PDAC. **Table S12.** Frequency of Core Gene Pathway Alterations according to Tumor Size in Age-related KRAS^mut^ PDAC. **Table S13.** Core Gene Pathway Alterations and Association with Distal Metastasis in Age-related PDAC. **Table S14.** Core Gene Alterations and Association with Tumor Differentiation in KRAS^mut^ PDAC in the TCGA cohort.

**Additional file 5: Figure S1.** Study design and flowchart of the study. **Figure S2.** Examples of the histological features of **A** well, **B** moderate and C poor differentiated PDAC. **Figure S3.** Illustration of how alterations of the Trithorax genes might result in the repression of TP53 function. **Figure S4.** The stepwise mode of PDAC carcinogenesis and relative genetic alterations.

## Data Availability

The data used and analysed in the study are available from the corresponding author on reasonable request.
