# Supplementary material for: TP53 missense mutation reveals gain-of-function properties in small-sized KRAS transformed pancreatic ductal adenocarcinoma
Source: J Transl Med. 2023 Dec 1;21:872. doi: 10.1186/s12967-023-04742-y (PMC10691048; doi:10.1186/s12967-023-04742-y)
Supplement: Supplementary file 4 — Additional file 4: Table S1–S14. Table S1. The Detailed Pathways and Genes Related to PDAC Carcinogenesis and Included in the Genetic Analyses Panel. Table S2. The siRNA Sequences and the Primer Sequences Used in the Study. Table S3. Patient Cohort and KRAS Status. Table S4. Clinical Information of the Patient Cohort. Table S5. Core Gene Pathway Alterations in KRASmut and KRASWT PDAC. Table S6. Core Gene Pathway Alterations and Association with Tumor Differentiation in age-related KRASmut PDAC. Table S7. Multivariate Logistic Regression of Core Gene Pathway Alterations for Tumor Differentiation in KRASmut PDAC. Table S8. Other Gene Pathway Alterations and Association with Lymph Nodes Involvement. Table S9. Frequency of Core Gene Pathway Alterations according to Tumor Size in KRASmut PDAC. Table S10. Other Gene Pathway Alterations and Association with Distal Metastasis. Table S11. Core Gene Pathway Alterations and Association with Lymph Nodes Involvement in Age-related PDAC. Table S12. Frequency of Core Gene Pathway Alterations according to Tumor Size in Age-related KRASmut PDAC. Table S13. Core Gene Pathway Alterations and Association with Distal Metastasis in Age-related PDAC. Table S14. Core Gene Alterations and Association with Tumor Differentiation in KRASmut PDAC in the TCGA cohort. [file 12967_2023_4742_MOESM4_ESM.docx]

Supplementary Table 1_The Detailed Pathways and Genes Related to PDAC Carcinogenesis and Included in the Genetic Analyses Panel

| Frequency | Pathway | Ralated genes |
| --- | --- | --- |
| High | RAS-MAPK pathway | KRAS, ALK, AXL, DDR2, ERBB1-4, FGFR1-4, KDR, KIT, MET, NTRK1-3, PDGFRA and PDGFRB, RET, SYK, CIC, HRAS, NRAS, NF1, ARAF, BRAF, RAF1, MAP2K1-2, MAP3K1, JAK1-3 |
| High | TP53 | TP53 |
| High | Cell cycle pathway (which is also downstream of TP53) | CDKN2A, CDKN2B, CDKN1B, RB1 |
| High | TGFβ pathway | SMAD4, SMAD2, SMAD3, TGFβR1, TGFβR2 |
| Low | Trithorax genes (which may also interlink with TP53 and cell cycle pathways) | 1. The COMPASS family: KDM6A, KMT2A, KMT2C, KMT2D  2. The SWI/SNF family: ARID1A, ARID1B, ARID2, CEBPA, PBRM1, SMARCA4, SMARCB1 |
| Low | Homologous recombination pathway | ARID1A which also belongs to the SWI/SNF family, ATM, BAP1, BARD1, BLM, BRCA1, BRCA2, BRIP1, CHEK2, FANCA, FANCC, NBN, PALB2, RAD50, RAD51C, RAD51D |
| Low | Mismatch repair pathway | MLH1, MSH2, MSH6, PMS2 |
| Low | RNA processing pathway | RBM10, SF3B1, U2AF1 |
| Low | PI3K-Akt pathway | PTEN, TSC1, TSC2 |
| Low | WNT pathway | APC, AXIN1, CTNNB1, FAT1, SOX9, RNF43 |
| Low | NOTCH pathway | NOTCH1, NOTCH2, NOTCH3, NOTCH4 |
| Low | Hedgehog pathway | PTCH1, SUFU |
| Low | DNA modification pathway | MUTYH, DNMT3A, DNMT3B, TET2 |

Supplementary Table 2_The siRNA Sequences and the Primer Sequences Used in the Study

| si-TP53_01 sense | CCACUGGAUGGAGAAUAUUTT |
| --- | --- |
| si-TP53_01 antisense | AAUAUUCUCCAUCCAGUGGTT |
| si-TP53_02 sense | CCAUCCACUACAACUACAUTT |
| si-TP53_02 antisense | AUGUAGUUGUAGUGGAUGGTT |
| TP53^R273H^ forward primer | CTGACTGTACCACCATCCACTAC |
| TP53^R273H^ reverse primer | CCAGGACAGGCACAAACATGC |
| TP53^C242R^ forward primer | CAACTACATGTGTAACAGTTCCCG |
| TP53^C242R^ reverse primer | TCAAAGCTGTTCCGTCCCAGTAG |

Supplementary Table 3_Patient Cohort and KRAS Status

|  | KRAS^mut^ | KRAS^WT^ |
| --- | --- | --- |
| PDAC without distal metastasis  Analysis of the resectable tumor  Biopsy of the unresectable tumor | 480  473  7 | 40  40  0 |
| PDAC with distal metastasis  Biopsy of the primary tumor  Biopsy of the distal metastasis | 115  49  66 | 4  2  2 |
| Total | 595 | 44 |

Supplementary Table 4_Clinical Information of the Patient Cohort

| Baseline characteristics of resectable cases | KRAS^mut^ (n=473) | KRAS^WT^ (n=40) |
| --- | --- | --- |
| Male | 285 (60.3%) | 24 (60.0%) |
| Median age (y) | 64 (28 - 84) | 64 (37 - 80) |
| Tumor location  Head/Uncinate  Neck  Body  Tail | 236 (49.9%)  33 (7.0%)  150 (31.7%)  54 (11.4%) | 20 (50.0%)  5 (12.5%)  14 (35.0%)  1 (2.5%) |
| Surgical type  Pancreaticoduodenectomy  Distal pancreatectomy  Appleby  Total pancreatectomy | 238 (50.3%)  170 (36.0%)  19 (4.0%)  46 (9.7%) | 23 (57.5%)  10 (25.0%)  3 (7.5%)  4 (10.0%) |
| Vascular invasion | 169 (35.7%) | 17 (42.5%) |
| Tumor differentiation  Moderate and well differentiated  Poor differentiated | 236 (49.9%)  237 (50.1%) | 24 (60.0%)  16 (40.0%) |
| pT stage  T1  T2  T3  T4 | 84 (17.8%)  302 (63.8%)  68 (14.4%)  19 (4.0%) | 9 (22.5%)  25 (62.5%)  3 (7.5%)  3 (7.5%) |
| pN stage  N0  N1  N2 | 225 (47.6%)  197 (41.6%)  51 (10.8%) | 23 (57.5%)  12 (30.0%)  5 (12.5%) |
| Resection margin status  R0  R1 | 395 (83.5%)  78 (16.5%) | 32 (80.0%)  8 (20.0%) |
| Perioperative death | 13 (2.7%) | 2 (5.0%) |
| Adjuvant chemotherapy | 329 (69.6%) | 23 (57.5%) |

Supplementary Table 5_Core Gene Pathway Alterations in KRAS^mut^ and KRAS^WT^ PDAC

| Mutated pathway | KRAS^mut^  n=595 | P | KRAS^WT^  n=44 | P |
| --- | --- | --- | --- | --- |
| MAPK | — | — | 14 (31.8%) | — |
| TP53  Missense  Truncating  Others^*^  Overall mutated | 256 (43.0%)  114 (19.2%)  13 (2.2%)  383 (64.4%) | 1  1  1  1 | 9 (20.5%)  2 (4.5%)  0  11 (25.0%) | **0.003^†^**  **0.015^†^**  —  **<0.001^†^** |
| Cell cycle | 110 (18.5%) | 1 | 3 (6.7%) | 0.050^†^ |
| TGFβ | 138 (23.2%) | 1 | 6 (13.6%) | 0.143^†^ |
| 2-3 mutated pathways^**^ | 171 (28.7%) | 1 | 5 (11.4%) | **0.013^†^** |
| Trithorax | 132 (22.2%) | 1 | 13 (29.5%) | 0.261^†^ |
| Subtype  Age-related  HRD  MMRD | 491 (82.5%)  92 (15.5%)  12 (2.0%) | 1  1  1 | 26 (59.1%)  17 (38.6%)  1 (2.3%) | **<0.001^†^**  **<0.001^†^**  0.908^‡^ |
| RNA processing | 19 (3.2%) | 1 | 0 | — |
| PI3K-Akt | 7 (1.2%) | 1 | 1 (2.3%) | 0.528^‡^ |
| WNT | 57 (9.6%) | 1 | 2 (4.5%) | 0.266^‡^ |
| NOTCH | 21 (3.5%) | 1 | 8 (18.2%) | **<0.001**^‡^ |
| Hedgehog | 14 (2.4%) | 1 | 4 (9.1%) | **0.009^‡^** |
| DNA modification | 19 (3.2%) | 1 | 4 (9.1%) | **0.043^‡^** |

^*^Other mutation subtypes included mixed cases, in-frame insertion or deletion and silent mutation; ^**^Alterations of 2-3 pathways among TP53, cell cycle pathway and TGFβ pathway; ^†^Chi-squared test; ^‡^Fisher exact test

Supplementary Table 6_Core Gene Pathway Alterations and Association with Tumor Differentiation in age-related KRAS^mut^ PDAC

| Mutated pathway in age-  related KRAS^mut^ PDAC | Moderate and well differentiated  n=215 | P | Poor differentiated  n=232 | P |
| --- | --- | --- | --- | --- |
| TP53  Missense  Truncating  Others^*^  Overall mutated | 86 (40.0%)  42 (19.5%)  1 (0.5%)  129 (60.0%) | 1  1  1  1 | 118 (50.9%)  47 (20.2%)  9 (3.9%)  174 (75.0%) | **0.021^†^**  0.848^†^  **<0.001^‡^**  **0.001^†^** |
| Cell cycle | 31 (14.4%) | 1 | 44 (19.0%) | 0.199^†^ |
| TGFβ | 50 (23.3%) | 1 | 45 (19.4%) | 0.319^†^ |
| 2-3 mutated pathways^**^ | 52 (24.2%) | 1 | 72 (31.0%) | 0.106^†^ |
| Trithorax | 30 (14.0%) | 1 | 38 (16.4%) | 0.476^†^ |
| RNA processing | 10 (4.7%) | 1 | 6 (2.6%) | 0.240^†^ |
| PI3K-Akt | 3 (1.4%) | 1 | 2 (0.9%) | 0.675^‡^ |
| WNT | 18 (8.4%) | 1 | 16 (6.9%) | 0.557^†^ |
| NOTCH | 4 (1.9%) | 1 | 9 (3.9%) | 0.204^†^ |
| Hedgehog | 6 (2.8%) | 1 | 6 (2.6%) | 0.894^†^ |
| DNA modification | 6 (2.8%) | 1 | 10 (4.3%) | 0.388^†^ |

^*^Other mutation subtypes included mixed cases, in-frame insertion or deletion and silent mutation; ^**^Alterations of 2-3 pathways among TP53, cell cycle pathway and TGFβ pathway; ^†^Chi-squared test; ^‡^Fisher exact test

Supplementary Table 7_Multivariate Logistic Regression of Core Gene Pathway Alterations for Tumor Differentiation in KRAS^mut^ PDAC

| Mutated pathway in  KRAS^mut^ PDAC | Moderate and well differentiated, n=260 | Poor differentiated  n=284 | OR [95% CI] | P |
| --- | --- | --- | --- | --- |
| TP53 missense | 94 (36.1%) | 144 (50.7%) | 1.848 [1.290 – 2.647] | **0.001** |
| Cell cycle | 42 (16.2%) | 58 (20.4%) | 1.190 [0.752 – 1.884] | 0.457 |
| TGFβ | 62 (23.8%) | 60 (21.1%) | 0.786 [0.519 – 1.191] | 0.257 |
| Trithorax | 52 (20.0%) | 70 (24.6%) | 1.184 [0.755 – 1.858] | 0.462 |
| HRD | 39 (15.0%) | 46 (16.2%) | 1.124 [0.670 – 1.886] | 0.657 |
| MMRD | 6 (2.3%) | 6 (2.1%) | 1.138 [0.342 – 3.785] | 0.834 |
| RNA processing | 11 (4.2%) | 8 (2.8%) | 0.680 [0.261 – 1.767] | 0.428 |
| PI3K-Akt | 3 (1.2%) | 3 (1.1%) | 0.652 [0.122 – 3.480] | 0.617 |
| WNT | 26 (10.0%) | 22 (7.7%) | 0.663 [0.357 – 1.234] | 0.195 |
| NOTCH | 10 (3.8%) | 10 (3.5%) | 0.846 [0.333 – 2.149] | 0.725 |
| Hedgehog | 7 (2.7%) | 7 (2.5%) | 1.028 [0.343 – 3.078] | 0.961 |
| DNA modification | 6 (2.3%) | 11 (3.9%) | 2.003 [0.710 – 5.647] | 0.189 |

Supplementary Table 8_Other Gene Pathway Alterations and Association with Lymph Nodes Involvement

| Mutated pathway | KRAS^mut^ | | KRAS^mut^ | | KRAS^mut^ | | KRAS^mut^ | | KRAS^WT^ | |
| --- | --- | --- | --- | --- | --- | --- | --- | --- | --- | --- |
|  | Overall, n=473 | | Tumor size ≤ 2cm, n=84 | | Tumor size (2, 3cm], n=211 | | Tumor size > 3cm/T4, n=178 | | Overall, n=40 | |
|  | N1-2 | P | N1-2 | P | N1-2 | P | N1-2 | P | N1-2 | P |
| HRD  WT  Mutated | 217/403 (53.8%)  31/70 (44.3%) | 1  0.139^†^ | 28/75 (37.3%)  3/9 (33.3%) | 1  0.814^‡^ | 104/176 (59.1%)  13/35 (37.1%) | 1  **0.017^†^** | 85/152 (55.9%)  15/26 (57.7%) | 1  0.866^†^ | 14/26 (53.8%)  3/14 (21.4%) | 1  **0.048^†^** |
| MMRD  WT  Mutated | 243/466 (52.1%)  5/7 (71.4%) | 1  0.454^‡^ | 31/84 (36.9%)  None | 1  — | 114/208 (54.8%)  3/3 | 1  0.255^‡^ | 98/174 (56.3%)  2/4 (50.0%) | 1  1.000^‡^ | 16/39 (41.0%)  1/1 | 1  — |
| RNA processing  WT  Mutated | 242/457 (53.0%)  6/16 (37.5%) | 1  0.224^†^ | 31/81 (38.3%)  0/3 | 1  — | 115/207 (55.6%)  2/4 (50.0%) | 1  1.000^‡^ | 96/169 (56.8%)  4/9 (44.4%) | 1  0.466^‡^ | 17/40 (42.5%)  None | 1  — |
| PI3K-Akt  WT  Mutated | 244/467 (52.2%)  4/6 (66.7%) | 1  0.688^‡^ | 30/83 (36.1%)  1/1 | 1  — | 116/210 (55.2%)  1/1 | 1  — | 98/174 (56.3%)  2/4 (50.0%) | 1  1.000^‡^ | 17/39 (43.6%)  0/1 | 1  — |
| NOTCH  WT  Mutated | 241/458 (52.6%)  7/15 (46.7%) | 1  0.650^†^ | 31/84 (36.9%)  None | 1  — | 113/205 (55.1%)  4/6 (66.7%) | 1  0.694^‡^ | 97/169 (57.4%)  3/9 (33.3%) | 1  0.156^‡^ | 15/33 (45.5%)  2/7 (28.6%) | 1  0.677^‡^ |
| DNA modification  WT  Mutated | 240/459 (52.3%)  8/14 (57.1%) | 1  0.720^†^ | 30/82 (36.6%)  1/2 (50.0%) | 1  1.000^‡^ | 114/205 (55.6%)  3/6 (50.0%) | 1  1.000^‡^ | 96/172 (55.8%)  4/6 (66.7%) | 1  0.697^‡^ | 16/36 (44.4%)  1/4 (25.0%) | 1  0.624^‡^ |

^†^Chi-squared test; ^‡^Fisher exact test

Supplementary Table 9_Frequency of Core Gene Pathway Alterations according to Tumor Size in KRAS^mut^ PDAC

| Mutated pathway in resected  KRAS^mut^ PDAC | Tumor size ≤ 2cm, n=84 | | Tumor size (2, 3cm], n=211 | | Tumor size > 3cm/T4, n=178 | |
| --- | --- | --- | --- | --- | --- | --- |
|  | Number | P | Number | P | Number | P |
| TP53  Missense  Truncating  Others^*^  Overall mutated | 31 (36.9%)  18 (21.4%)  1 (1.2%)  50 (59.5%) | 1  1  1  1 | 80 (37.9%)  46 (21.8%)  6 (2.8%)  132 (62.5%) | 0.872^†^  0.944^†^  0.400^‡^  0.628^†^ | 86 (48.3%)  29 (16.3%)  4 (2.2%)  119 (66.8%) | 0.083^†^  0.312^†^  1.000^‡^  0.247^†^ |
| Cell cycle | 12 (14.3%) | 1 | 41 (19.4%) | 0.299^†^ | 33 (18.5%) | 0.394^†^ |
| TGFβ | 12 (14.3%) | 1 | 41 (19.4%) | 0.299^†^ | 46 (25.8%) | **0.035^†^** |
| 2-3 mutated pathways^**^ | 16 (19.0%) | 1 | 59 (28.0%) | 0.113^†^ | 54 (30.3%) | 0.054^†^ |
| Trithorax | 9 (10.7%) | 1 | 45 (21.35) | **0.033^†^** | 45 (25.3%) | **0.007^†^** |
| HRD | 9 (10.7%) | 1 | 35 (16.6%) | 0.201^†^ | 26 (14.6%) | 0.387^†^ |
| MMRD | None | 1 | 3 (1.4%) | 0.561^‡^ | 4 (2.2%) | 0.309^‡^ |

^*^Other mutation subtypes included mixed cases, in-frame insertion or deletion and silent mutation; ^**^Alterations of 2-3 pathways among TP53, cell cycle pathway and TGFβ pathway; ^†^Chi-squared test; ^‡^Fisher exact test

Supplementary Table 10_Other Gene Pathway Alterations and Association with Distal Metastasis

| Mutated pathway | KRAS^mut^ | | KRAS^mut^ | | KRAS^mut^ | | KRAS^mut^ | | KRAS^WT^ | |
| --- | --- | --- | --- | --- | --- | --- | --- | --- | --- | --- |
|  | Overall, n=595 | | Tumor size ≤ 2cm, n=96 | | Tumor size (2, 3cm], n=261 | | Tumor size > 3cm/T4, n=238 | | Overall, n=44 | |
|  | Metastasis | P | Metastasis | P | Metastasis | P | Metastasis | P | Metastasis | P |
| RNA processing  WT  Mutated | 112/576 (19.4%)  3/19 (15.8%) | 1  0.691^‡^ | 11/92 (12.0%)  1/4 (25.0%) | 1  0.419^‡^ | 48/255 (18.8%)  2/6 (33.3%) | 1  0.324^‡^ | 53/229 (23.1%)  0/9 | 1  — | 4/44 (9.1%)  None | 1  — |
| PI3K-Akt  WT  Mutated | 114/588 (19.4%)  1/7 (14.3%) | 1  0.734^‡^ | 12/95 (12.6%)  0/1 | 1  — | 49/259 (22.8%)  1/2 (50.0%) | 1  0.347^‡^ | 53/234 (22.6%)  0/4 | 1  — | 4/43 (9.3%)  0/1 | 1  — |
| WNT  WT  Mutated | 101/538 (18.8%)  14/57 (24.6%) | 1  0.293^†^ | 11/89 (12.4%)  1/7 (14.3%) | 1  0.882^‡^ | 42/229 (18.3%)  8/32 (25.0%) | 1  0.370^†^ | 48/220 (12.8%)  5/18 (27.8%) | 1  0.559^‡^ | 4/42 (9.5%)  0/2 | 1  — |
| NOTCH  WT  Mutated | 109/574 (19.0%)  6/21 (28.6%) | 1  0.275^‡^ | 11/95 (11.6%)  1/1 | 1  — | 47/252 (18.7%)  3/9 (33.3%) | 1  0.271^‡^ | 51/227 (22.5%)  2/11 (18.2%) | 1  0.739^‡^ | 3/36 (8.3%)  1/8 | 1  0.566^‡^ |
| Hedgehog  WT  Mutated | 113/581 (19.4%)  2/14 (14.35) | 1  0.629^‡^ | 12/93 (12.9%)  0/3 | 1  — | 49/253 (11.5%)  1/8 (12.5%) | 1  0.627^‡^ | 52/235 (22.1%)  1/3 (33.3%) | 1  0.532^‡^ | 3/40 (7.5%)  1/4 (25.0%) | 1  0.327^‡^ |
| DNA modification  WT  Mutated | 111/576 (19.3%)  4/19 (21.1%) | 1  0.847^‡^ | 12/94 (12.8%)  0/2 | 1  — | 49/254 (19.3%)  1/7 (14.3%) | 1  0.740^‡^ | 50/228 (21.9%)  3/10 (30.0%) | 1  0.548^‡^ | 4/40 (10.0%)  0/4 | 1  — |

^†^Chi-squared test; ^‡^Fisher exact test

Supplementary Table 11_Core Gene Pathway Alterations and Association with Lymph Nodes Involvement in Age-related PDAC

| Mutated pathway | KRAS^mut^ | | KRAS^mut^ | | KRAS^mut^ | | KRAS^mut^ | | KRAS^WT^ | |
| --- | --- | --- | --- | --- | --- | --- | --- | --- | --- | --- |
|  | Overall age-related, n=396 | | Tumor size ≤ 2cm, n=75 | | Tumor size (2, 3cm], n=173 | | Tumor size > 3cm/T4, n=148 | | Age-related, n=25 | |
|  | N1-2 | P | N1-2 | P | N1-2 | P | N1-2 | P | N1-2 | P |
| TP53  WT  Missense  Truncating  Others^*^  Mutated | 72/135 (53.3%)  93/174 (53.4%)  42/79 (53.2%)  5/8 (62.5%)  140/261 (53.6%) | 1  0.984^†^  0.981^†^  0.725^‡^  0.954^†^ | 8/31 (25.8%)  15/28 (53.6%)  4/15 (26.7%)  1/1  20/44 (45.5%) | 1  **0.029^†^**  0.950^‡^  —  0.083^†^ | 38/64 (59.4%)  39/67 (58.2%)  21/37 (57.8%)  3/5 (60.0%)  63/109 (57.8%) | 1  0.892^†^  0.797^†^  1.000^‡^  0.893^†^ | 26/40 (65.0%)  39/79 (49.4%)  17/27 (63.0%)  1/2 (50.0%)  57/108 (52.8%) | 1  0.106^†^  0.865^†^  1.000^‡^  0.183^†^ | 10/18 (55.6%)  2/6 (33.3%)  1/1  None  3/7 (42.9%) | 1  0.640^‡^  —  —  0.673^‡^ |
| Cell cycle  WT  Mutated | 177/331 (53.5%)  35/65 (53.8%) | 1  0.956^†^ | 23/64 (50.0%)  5/11 (45.5%) | 1  0.547^‡^ | 82/143 (57.3%)  19/30 (63.3%) | 1  0.545^†^ | 72/124 (58.1%)  11/24 (45.8%) | 1  0.269^†^ | 11/22 (50.0%)  2/3 (66.7%) | 1  1.000^‡^ |
| TGFβ  WT  Mutated | 166/315 (52.7%)  46/81 (56.8%) | 1  0.510^†^ | 23/64 (35.9%)  5/11 (45.5%) | 1  0.547^‡^ | 80/138 (58.0%)  21/35 (60.0%) | 1  0.828^†^ | 63/113 (55.8%)  20/35 (57.1%) | 1  0.885^†^ | 11/21 (52.4%)  2/4 (50.0%) | 1  1.000^‡^ |
| Mutated pathways  0-1  2-3^**^ | 154/290 (53.1%)  58/106 (54.7%) | 1  0.776^†^ | 20/61 (32.8%)  8/14 (57.1%) | 1  0.089^†^ | 73/127 (57.5%)  28/46 (60.9%) | 1  0.689^†^ | 61/102 (59.8%)  22/46 (47.8%) | 1  0.174^†^ | 12/21 (57.1%)  1/4 (25.0%) | 1  0.322^‡^ |
| Trithorax  WT  Mutated | 186/341 (54.5%)  26/55 (47.3%) | 1  0.316^†^ | 25/70 (35.7%)  3/5 (60.0%) | 1  0.356^‡^ | 92/150 (61.3%)  9/23 (39.1%) | 1  **0.044^†^** | 69/121 (57.0%)  14/27 (51.9%) | 1  0.624^†^ | 9/19 (47.4%)  4/6 (66.7%) | 1  0.645^‡^ |

^*^Other mutation subtypes included mixed cases, in-frame insertion or deletion and silent mutation; ^**^Alterations of 2-3 pathways among TP53, cell cycle pathway and TGFβ pathway; ^†^Chi-squared test; ^‡^Fisher exact test

Supplementary Table 12_Frequency of Core Gene Pathway Alterations according to Tumor Size in Age-related KRAS^mut^ PDAC

| Mutated pathway in resected  age-related KRAS^mut^ PDAC | Tumor size ≤ 2cm, n=75 | | Tumor size (2, 3cm], n=173 | | Tumor size > 3cm/T4, n=148 | |
| --- | --- | --- | --- | --- | --- | --- |
|  | Number | P | Number | P | Number | P |
| TP53  Missense  Truncating  Others  Overall mutated | 28 (37.3%)  15 (20.0%)  1 (1.3%)  44 (58.6%) | 1  1  1  1 | 67 (38.7%)  37 (21.4%)  5 (2.9%)  109 (63.0%) | 0.836^†^  0.805^†^  0.671^‡^  0.519^†^ | 79 (53.4%)  27 (18.2%)  2 (1.4%)  108 (73.0%) | **0.023^†^**  0.751^†^  1.000^‡^  **0.003^†^** |
| Cell cycle | 11 (14.7%) | 1 | 30 (17.3%) | 0.603^†^ | 24 (16.2%) | 0.764^†^ |
| TGFβ | 11 (14.7%) | 1 | 35 (20.2%) | 0.300^†^ | 35 (23.6%) | 0.117^†^ |
| 2-3 mutated pathways | 14 (18.7%) | 1 | 46 (26.6%) | **0.040^†^** | 46 (31.1%) | **0.008^†^** |
| Trithorax | 5 (6.7%) | 1 | 23 (13.3%) | 0.130^†^ | 27 (18.2%) | **0.020^†^** |

^†^Chi-squared test; ^‡^Fisher exact test

Supplementary Table 13_Core Gene Pathway Alterations and Association with Distal Metastasis in Age-related PDAC

| Mutated pathway | KRAS^mut^ | | KRAS^mut^ | | KRAS^mut^ | | KRAS^mut^ | | KRAS^WT^ | |
| --- | --- | --- | --- | --- | --- | --- | --- | --- | --- | --- |
|  | Overall age-related, n=491 | | Tumor size ≤ 2cm, n=83 | | Tumor size (2, 3cm], n=215 | | Tumor size > 3cm/T4, n=193 | | Age-related, n=26 | |
|  | Metastasis | P | Metastasis | P | Metastasis | P | Metastasis | P | Metastasis | P |
| TP53  WT  Missense  Truncating  Others^*^  Mutated | 27/164 (16.5%)  45/222 (20.3%)  17/96 (17.7%)  1/9 (11.1%)  63/327 (19.3%) | 1  0.343^†^  0.796^†^  0.671^‡^  0.449^†^ | 0/31  6/34 (17.6%)  1/16 (6.3%)  1/2  8/52 (15.4%) | 1  **0.025^‡^**  0.340^‡^  —  **0.022^‡^** | 14/78 (17.9%)  18/85 (21.2%)  9/46 (19.6%)  1/6 (16.7%)  28/137 (20.4%) | 1  0.604^†^  0.823^†^  1.000^‡^  0.658^†^ | 13/55 (23.6%)  20/102 (19.6%)  7/34 (20.6%)  0/2  27/138 (19.6%) | 1  0.555^†^  0.738^†^  —  0.529^†^ | 1/19 (5.3%)  0/6  0/1  None  0/7 | 1  —  —  —  — |
| Cell cycle  WT  Mutated | 74/409 (18.1%)  16/82 (19.5%) | 1  0.762^†^ | 7/71 (9.9%)  1/12 (8.3%) | 1  0.554^‡^ | 32/175 (18.3%)  10/40 (25.0%) | 1  0.334^†^ | 35/163 (21.5%)  5/30 (16.7%) | 1  0.551^†^ | 1/23 (4.3%)  0/3 | 1  — |
| TGFβ  WT  Mutated | 66/385 (17.1%)  24/106 (22.6%) | 1  0.195^†^ | 6/70 (8.6%)  2/13 (15.4%) | 1  0.445^‡^ | 31/169 (18.3%)  11/46 (23.9%) | 1  0.398^†^ | 29/146 (19.9%)  11/47 (23.4%) | 1  0.602^†^ | 1/22 (4.5%)  0/4 | 1  — |
| Mutated pathways  0-1  2-3^**^ | 60/353 (17.0%)  30/138 (16.4%) | 1  0.222^†^ | 5/66 (7.6%)  3/17 (17.6%) | 1  0.210^‡^ | 27/154 (17.5%)  15/61 (24.6%) | 1  0.239^†^ | 28/133 (21.1%)  12/60 (20.0%) | 1  0.867^†^ | 1/22 (4.5%)  0/4 | 1  — |
| Trithorax  WT  Mutated | 72/416 (17.3%)  18/75 (24.0%) | 1  0.168^†^ | 7/77 (9.1%)  1/6 (16.7%) | 1  0.545^‡^ | 31/181 (17.1%)  11/34 (32.4%) | 1  **0.040^†^** | 34/158 (21.5%)  6/35 (17.1%) | 1  0.563^†^ | 0/19  1/7 (14.3%) | 1  — |

^*^Other mutation subtypes included mixed cases, in-frame insertion or deletion and silent mutation; ^**^Alterations of 2-3 pathways among TP53, cell cycle pathway and TGFβ pathway; ^†^Chi-squared test; ^‡^Fisher exact test

Supplementary Table 14_Core Gene Alterations and Association with Tumor Differentiation in KRAS^mut^ PDAC in the TCGA cohort

| Mutated genes | Well differentiated  n=11 | P | Moderate differentiated  n=50 | P | Poor differentiated  n=30 | P |
| --- | --- | --- | --- | --- | --- | --- |
| TP53  Missense  Truncating | 0 (0%)  8 (72.7%) | 1  1 | 27 (54.0%)  10 (20.0%) | **0.001^‡^**  **0.001^‡^** | 14 (46.7%)  10 (33.3%) | **0.007^‡^**  **0.036^‡^** |
| CDKN2A | 3 (27.3%) | 1 | 11 (22.0%) | 0.703^‡^ | 9 (30.0%) | 1.000^‡^ |
| SMAD4 | 1 (9.1%) | 1 | 17 (34.0%) | 0.150^‡^ | 7 (23.3%) | 0.412^‡^ |

^†^Chi-squared test; ^‡^Fisher exact test
