# Supplementary material for: TP53 missense mutation reveals gain-of-function properties in small-sized KRAS transformed pancreatic ductal adenocarcinoma
Source: J Transl Med. 2023 Dec 1;21:872. doi: 10.1186/s12967-023-04742-y (PMC10691048; doi:10.1186/s12967-023-04742-y)
Supplement: Supplementary file 5 — Additional file 5: Figure S1. Study design and flowchart of the study. Figure S2. Examples of the histological features of A well, B moderate and C poor differentiated PDAC. Figure S3. Illustration of how alterations of the Trithorax genes might result in the repression of TP53 function. Figure S4. The stepwise mode of PDAC carcinogenesis and relative genetic alterations. [file 12967_2023_4742_MOESM5_ESM.docx]

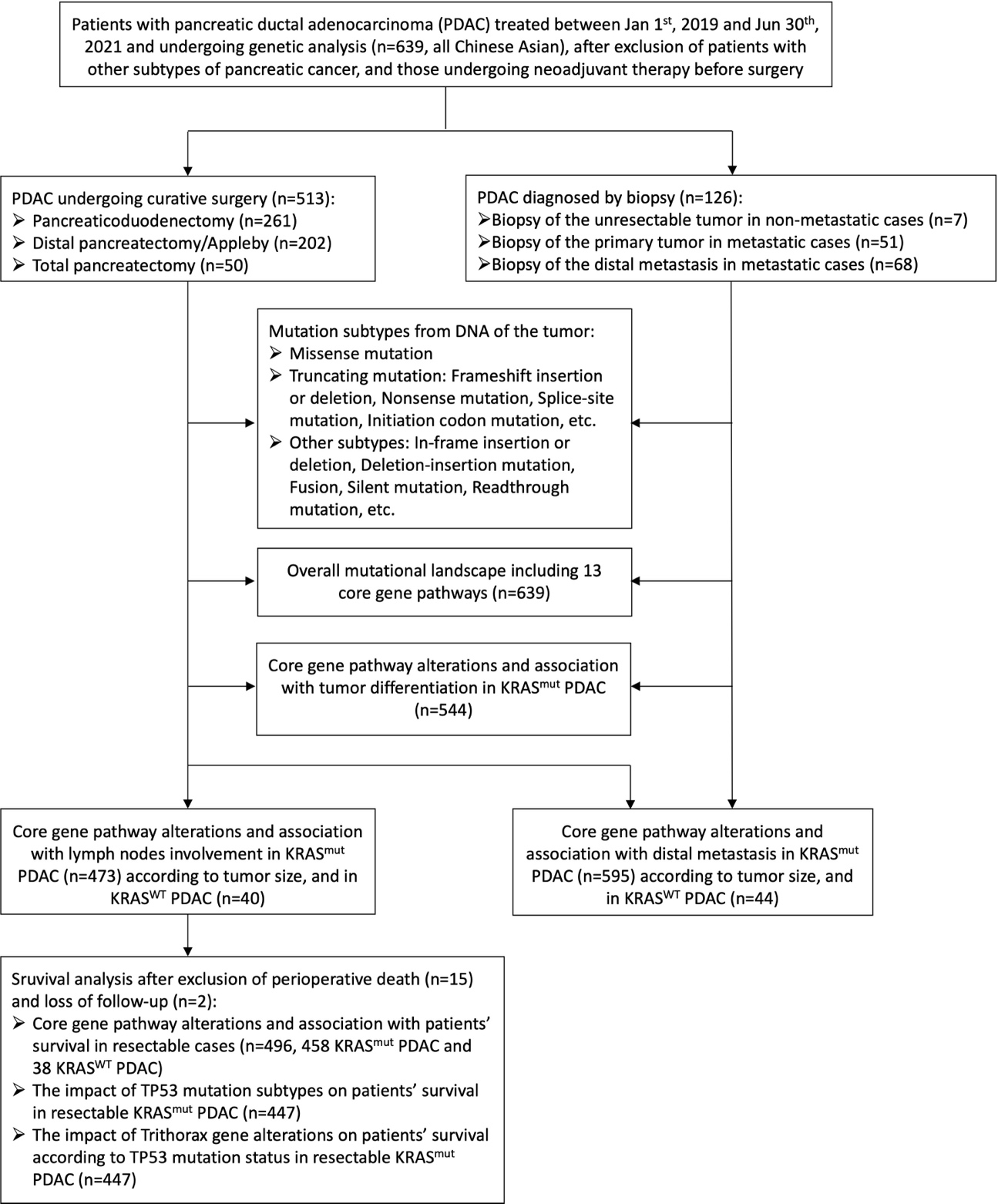


Supplementary Figure 1_Study design and flowchart of the study


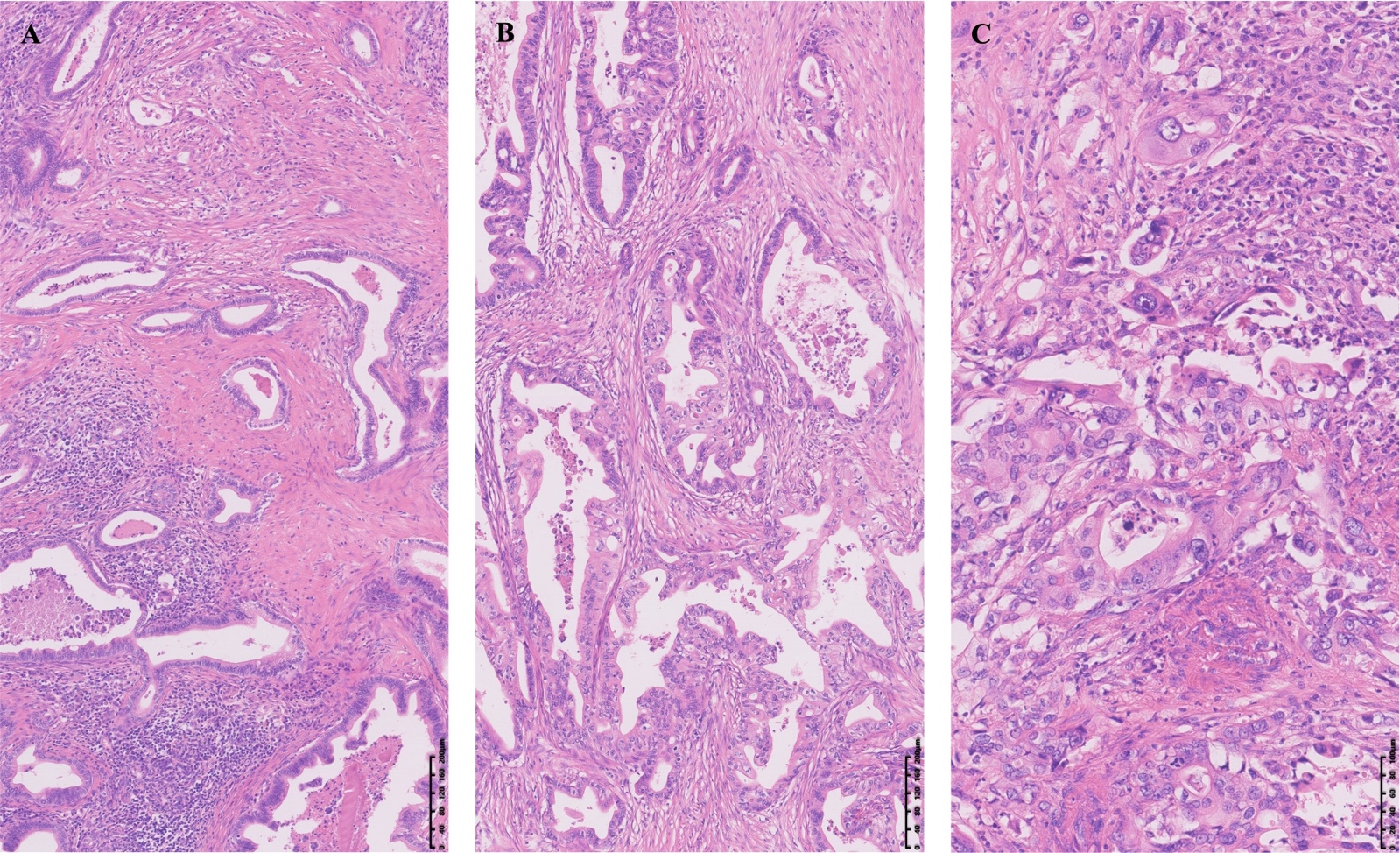


Supplementary Figure 2_Examples of the histological features of (A) well, (B) moderate and (C) poor differentiated PDAC


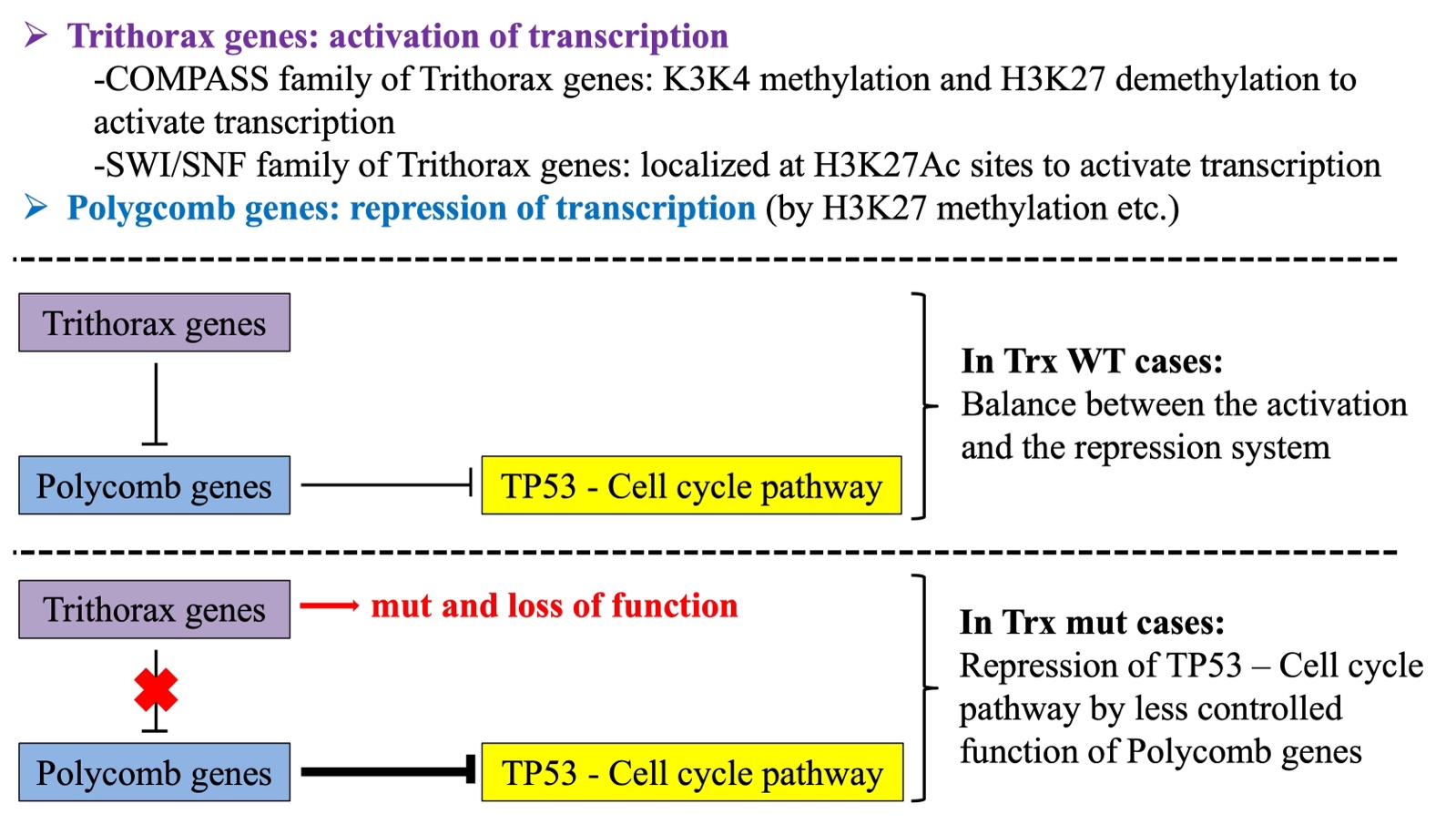


Supplementary Figure 3_Illustration of how alterations of the Trithorax genes might result in the repression of TP53 function


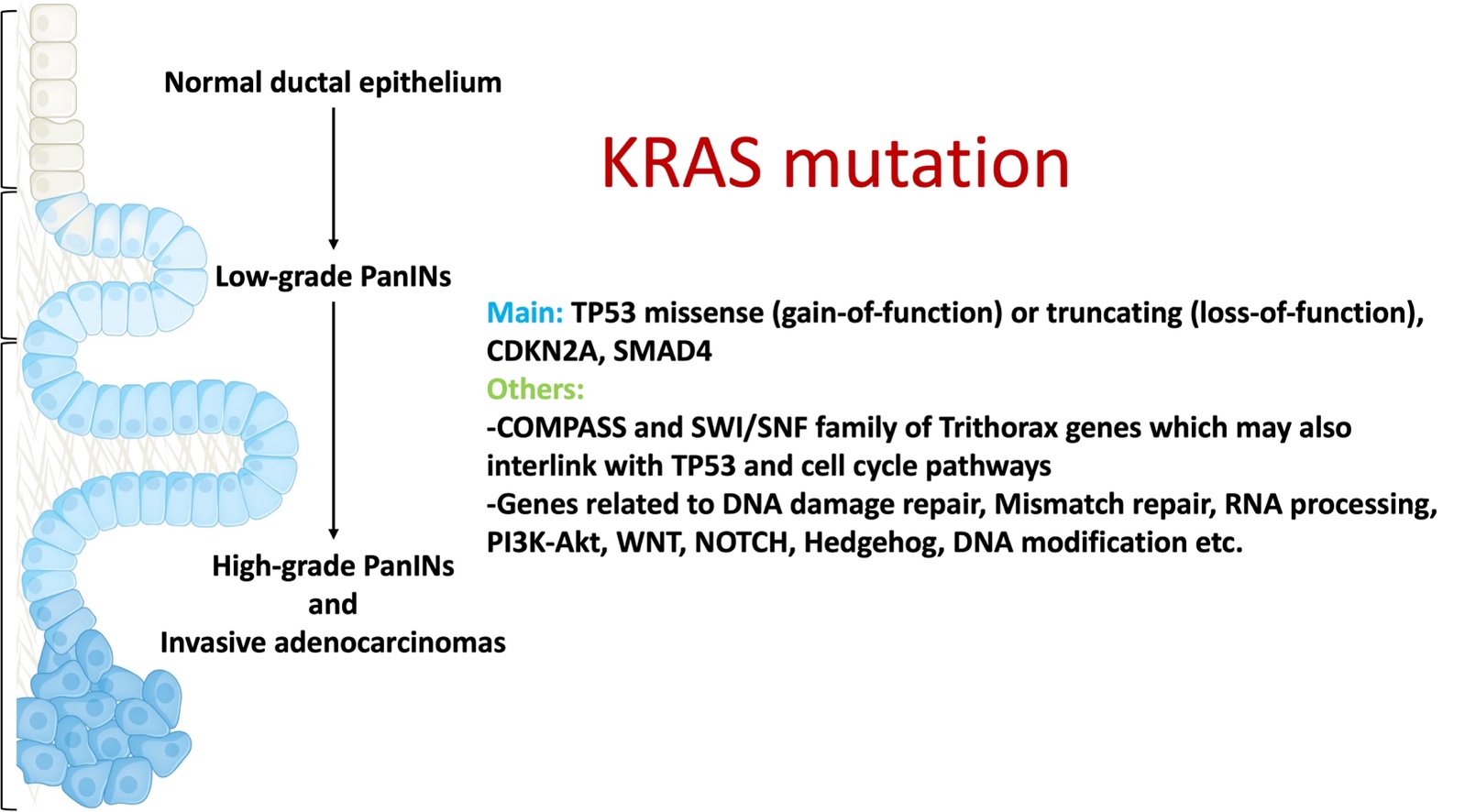


Supplementary Figure 4_The stepwise mode of PDAC carcinogenesis and relative genetic alterations
